# Degradable and Recyclable 3D‐Printed Pheromones Delivery System Reinforced by Metal Coordination Cross‐Linking for Efficient Pest Trapping

**DOI:** 10.1002/advs.202509712

**Published:** 2025-11-29

**Authors:** Teng Wang, Wenjie Shangguan, Fang Zhang, Wenlong Liang, Frederik R. Wurm, Qiliang Huang, Lidong Cao

**Affiliations:** ^1^ Key Laboratory of Integrated Pest Management in Crops Institute of Plant Protection Chinese Academy of Agricultural Sciences Beijing 100193 P. R. China; ^2^ College of Chemistry and Life Science Beijing University of Technology Beijing 100124 P. R. China; ^3^ Sustainable Polymer Chemistry Department of Molecules and Materials MESA+Institute for Nanotechnology Faculty of Science and Technology Universiteit Twente PO Box 217 Enschede 7500 AE The Netherlands

**Keywords:** 3D printing, green agriculture, insect sex pheromones, metal coordination, recyclable materials

## Abstract

Prompted by health and environmental concerns over chemical pesticides, insect sex pheromones offer a promising eco‐friendly pest control alternative. However, their high volatility and degradability cause instability, which hinders widespread use. Currently, most pheromone carriers (e.g., commercial rubber) suffer from short release cycles, non‐degradability, and non‐recyclability, creating a need for sustainable alternatives. The study developed an insect pheromone sustained‐release carrier using a cellulose acetate matrix fabricated via direct ink writing. The incorporation of lignin sulfonate and Fe^3+^ ions improved the carrier's mechanical strength and release performance. The 3D‐printed carriers achieved over 90% encapsulation efficiency and provided sustained release for up to six weeks. Compared to commercial rubber carriers, they exhibit higher cumulative trapping effectiveness against *Grapholita molesta*, catching 53 ± 6 insects over six weeks, versus 38 ± 4 for the rubber carrier. They also demonstrate a soil cumulative degradation rate of 20.4 ± 0.8% within 105 days, indicating environmental sustainability. Notably, the carrier can be physically recycled and reprinted. The reprinted carrier maintains an encapsulation efficiency of 92.8 ± 3.4% and bioactivity. This novel 3D‐printed system combines rapid production, degradability, and recyclability, offering a sustainable and efficient alternative to conventional pest control and supporting green agricultural innovation.

## Graphical abstract

This study presents a 3D‐printed carrier for the slow release of insect pheromones. Reinforced by lignosulfonate and Fe³⁺ coordination in a cellulose acetate matrix, it enables sustained release for up to six weeks with remarkable trapping efficacy. Featuring soil degradability and recyclable re‐printing capability, this carrier provides a sustainable pest control solution for green agriculture.

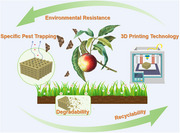

## 1 Introduction

As the global population continues to grow, the demand for food has risen significantly. Traditionally, crop pest management has heavily relied on chemical pesticides, which are often ecologically harmful and unsustainable. In recent years, the extensive use of chemical pesticides has led to several critical issues, including the gradual development of pest resistance and severe contamination of soil and water resources.^[^ ^1^ ^]^ Insect sex pheromones that induce physiological responses in insects offer a promising alternative for efficient pest management.^[^ ^2^, ^3^ ^]^ Strategies utilizing insect sex pheromones for pest control include monitoring, mass trapping, attract‐and‐kill, mating disruption, and push–pull strategy (stimulo‐deterrent diversion).^[^ ^4^ ^]^ These chemicals are highly species‐specific, targeting only the intended pests, and remain effective even in minute quantities.^[^ ^5^ ^]^ However, most pheromone components are volatile and tend to dissipate quickly unless incorporated into controlled‐release devices.^[^ ^6^ ^]^ Current pheromone delivery systems can be broadly categorized into traditional release devices and novel functional materials. Conventional carriers such as rubber septa, plastic tubes, and laminated films offer advantages like low cost and well‐established manufacturing processes. However, they predominantly rely on solvent‐assisted physical adsorption of pheromones, often resulting in short release durations, poor degradability, and a need for frequent replacement, which limits their suitability for long‐lasting and simplified pest control in open‐field applications.^[^ ^7^ ^]^ To overcome the limitations of traditional systems, recent research has developed novel carriers including electrospun fibers, nanocapsules, and graphene oxide composites, which have shown progress in terms of release controllability, loading capacity, and environmental compatibility.^[^ ^8^, ^9^, ^10^ ^]^ Nevertheless, these advanced materials often face challenges such as complex preparation processes and difficulties in scaling up production. Consequently, a trade‐off commonly exists between performance and practicality, and an ideal system that simultaneously achieves durable efficacy, controllable release, environmental safety, and manufacturing feasibility has yet to be realized.^[^ ^11^, ^12^ ^]^ Therefore, developing novel carrier construction technologies that combine controllable release characteristics with good scalability is crucial for advancing the practical application of pheromone‐based control techniques. In this context, this study proposes the use of 3D printing technology to fabricate structured pheromone carriers. By functionalizing composite printing materials and precisely tailoring the carrier architecture, this strategy enables programmable control of pheromone release behavior, providing a promising route to harmonize the competing demands of longevity, controllability, and scalable production in sustainable pest management systems.

3D printing, recognized as a transformative additive manufacturing technology, has demonstrated revolutionary potential across interdisciplinary domains including biomedical engineering, architecture, and advanced materials development.^[^ ^13^, ^14^, ^15^, ^16^ ^]^ Distinct from conventional subtractive manufacturing, this technology enables resource‐efficient fabrication of intricate 3D architectures through streamlined workflows, while providing unprecedented design flexibility and rapid iteration capabilities.^[^ ^17^ ^]^ The convergence of 3D printing with polymer composites has particularly catalyzed material innovation, addressing performance constraints and expanding application frontiers through programmable material‐structure integration.^[^ ^18^ ^]^ Among diverse 3D printing technologies such as fused deposition modeling^[^ ^19^ ^]^ and stereolithography,^[^ ^20^ ^]^ direct ink writing (DIW) emerges as a particularly versatile platform for polymer composite engineering. This ambient‐temperature extrusion‐based technique eliminates requirements for photopolymerization or thermal processing, thereby preserving functional additives and volatile components while enabling customized ink formulations with multifunctional enhancements.^[^ ^21^, ^22^ ^]^ In this study, DIW was selected as the fabrication technique because it uniquely satisfies the processing requirements of pheromone‐based delivery systems. Unlike FDM, which involves high extrusion temperatures, or SLA, which relies on UV irradiation and photoinitiators, DIW operates under ambient or low‐temperature conditions, thereby preserving the bioactivity of thermally and photosensitive compounds such as pheromones. Furthermore, DIW exhibits excellent material versatility, allowing the formulation of composite inks containing functional additives such as metal ion crosslinkers, biodegradable polymers, or stimulus‐responsive components. This compatibility enables precise tuning of rheological properties and molecular interactions, which is critical for achieving programmable and sustained release behavior. Such operational advantages position DIW as a pivotal methodology for designing functional composites with tailored physicochemical properties. Despite its widespread use in other industries, the application of 3D printing in agricultural plant protection remains underexplored. Specifically, the development of insect pheromone carriers through 3D printing presents a promising opportunity to enable eco‐friendly, efficient pesticide application. This innovation could significantly advance green pesticide solutions and open new research avenues in integrated pest management.

Cellulose, one of the most abundant natural polymers, is increasingly being explored as a sustainable alternative to petroleum‐based plastics in 3D printing.^[^ ^23^ ^]^ It offers numerous advantages, including biodegradability, biocompatibility, non‐toxicity, and recyclability, making it an ideal candidate for eco‐friendly printing materials.^[^ ^24^ ^]^ Cellulose acetate (CA), as an important derivative of cellulose, has been investigated for applications in green functional carriers due to its good biocompatibility, ease of processing, and high loading capacity for active molecules. Examples include preparing pheromone‐sustained release nanofibers via electrospinning^[^ ^25^ ^]^ or developing composite films for intelligent monitoring and packaging.^[^ ^26^ ^]^ However, these studies still face certain limitations, such as insufficient mechanical strength, poor environmental durability, and an incomplete assessment of their full lifecycle sustainability, particularly recyclability. To address these shortcomings, 3D printing technology offers distinct advantages. First, it allows for the continuation of material functionalization, such as incorporating specific components to compensate for the weaknesses of single materials. More importantly, through the unique design of controllable matrix structures enabled by 3D printing, carriers can be optimized in terms of mechanical properties, environmental durability, and UV resistance, making them more capable of withstanding complex and harsh field conditions. Additionally, leveraging the efficiency and rapid prototyping capabilities of 3D printing facilitates the quick preparation, iterative optimization, and cyclic recycling of carriers. However, to meet the high‐performance requirements of commercial 3D‐printed products, cellulose‐based materials often require further modification with natural or chemical additives, such as the incorporation of metal ions as cross‐linking agents. The chelation effect between metal ions and biopolymers forms three‐dimensional structures, which can improve the mechanical properties of the composite.^[^ ^27^ ^]^ These materials provide valuable opportunities to produce 3D objects with tailored properties from inexpensive and renewable bioresources. This study developed cellulose acetate‐based printing inks incorporating lignin sulfonate and Fe^3+^, and then used DIW to fabricate delivery carriers loaded with insect sex pheromones of *Grapholita molesta* (Lepidoptera: Tortricidae) (GmSP). Beyond examining the printability of ink, we thoroughly investigated key properties of the 3D‐printed carrier, including its chemical composition, surface morphology, physical properties, encapsulation and release characteristics, trapping efficiency, degradability, and recyclability. The findings of this research provide novel insights into the development and application of eco‐sustainable carriers for insect pheromones, contributing to the advancement of green agriculture.

## 2 Results and Discussion

### 2.1 Design and Fabrication of 3D‐Printed Pheromones Delivery System

**Figure 1:**
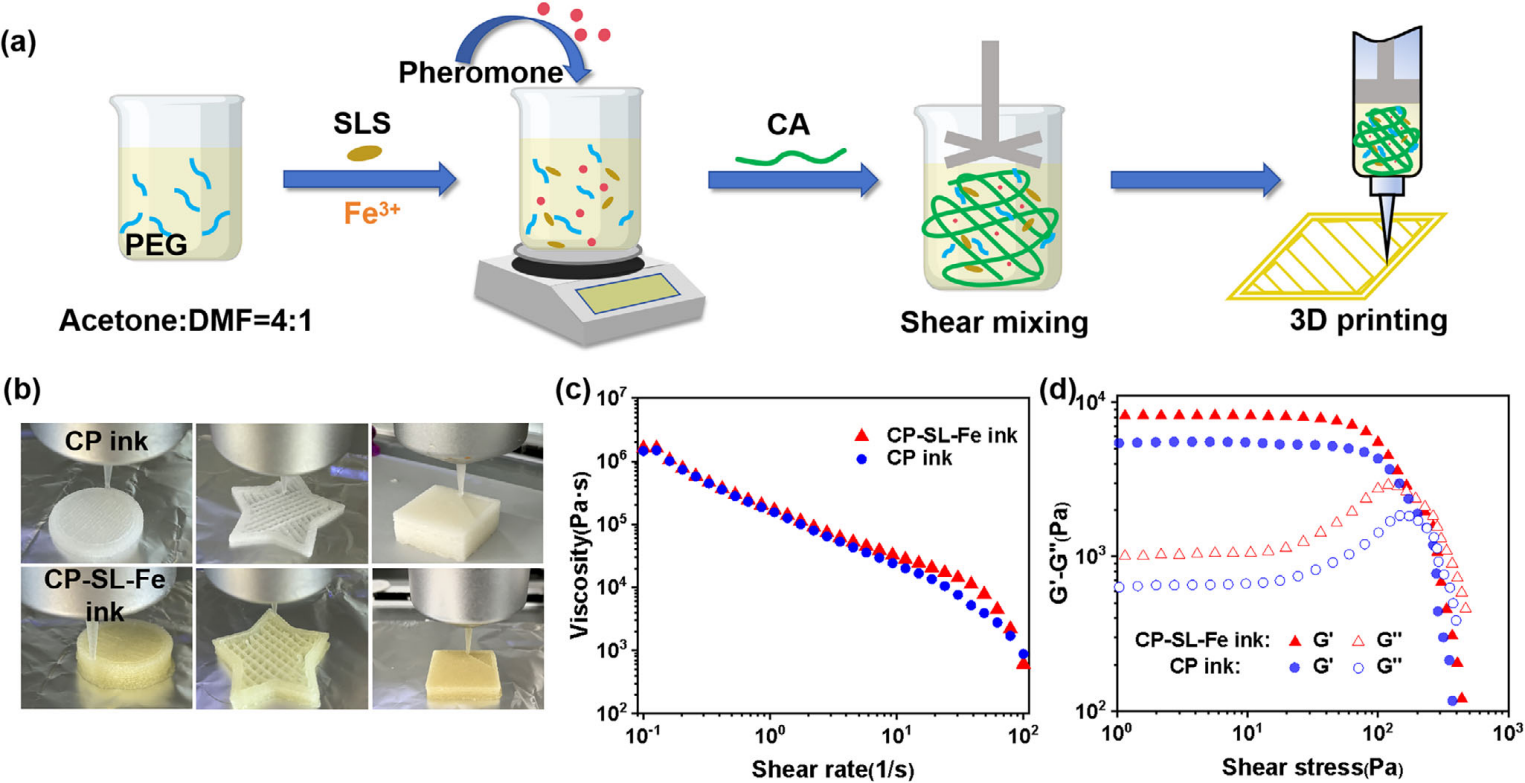
Preparation and rheological characterization of 3D printing inks. a) Schematic diagram of the ink preparation process. b) Photographs of the extrusion molding of CP and CP‐SL‐Fe inks. c) Viscosity as a function of the shear rate of two inks. d) Storage modulus (G′) and loss modulus (G″) versus oscillation stress.

In this study, a one‐step methodology was employed to fabricate pheromone‐loaded 3D printing inks, enabling direct ink writing at room temperature, as illustrated in **Figure** **1**a. The ink formulation utilized a binary solvent system comprising acetone and dimethylformamide (DMF). The base ink consisted of CA, polyethylene glycol (PEG), and a certain amount of pheromone, designated as CP ink. Subsequent incorporation of sodium lignosulfonate (SLS) and ferric ions (FeCl_3_) yielded the modified ink, designated as CP‐SL‐Fe ink. Current literature demonstrates that PEG functions effectively as a plasticizer in 3D printing inks,^[^ ^28^ ^]^ while lignin derivatives exhibit superior UV‐blocking capabilities, serving as effective reinforcing agents in polymer matrices.^[^ ^29^ ^]^ Furthermore, the abundant hydroxyl groups present in the polymer matrix facilitate the formation of coordination bonds with Fe^3+^ ions, promoting complex crosslinking networks that significantly enhance the overall performance of 3D‐printed carriers.^[^ ^30^ ^]^ Figure 1b illustrated the extrusion process of the two ink formulations, which can easily print geometric shapes such as cylinders, pentagons, and cubes. The experimental results demonstrated that both ink formulations exhibited satisfactory printability and shape retention. The extruded ink undergone a rapid phase transition from semi‐solid to solid state upon nozzle deposition, forming mechanically stable filaments that maintain structural integrity in ambient conditions. The gradient volatility of the binary solvent system facilitates the smooth progression of the process.^[^ ^31^ ^]^ The high volatility of acetone facilitates rapid ink solidification at room temperature, ensuring dimensional stability during the printing process. Concurrently, the residual low‐volatility DMF solvent maintains appropriate ink fluidity, ensuring continuous extrusion and effective inter‐filament bonding throughout the printing process. Following the printing process, the residual solvents can be removed by drying to accelerate the shaping of the carrier.^[^ ^32^ ^]^

### 2.2 Rheological Characterization of Pheromone‐Loaded Inks

To ensure the ink's printability, the rheological properties of CP and CP‐SL‐Fe were analyzed. Both CP and CP‐SL‐Fe demonstrated distinct shear‐thinning behavior with increasing shear rate (Figure 1c). The viscosity measurements revealed an increase from 1.4±0.04 MPa∙s for the CP ink to 1.6±0.003 MPa∙s for the CP‐SL‐Fe ink at a shear rate of 0.1 s^−1^. The introduction of SLS and Fe^3+^ led to a slight increase in the viscosity of the modified ink, which can be attributed to the non‐covalent interactions between the functional groups of CA, SLS and Fe^3+^. Such shear‐thinning behavior is particularly essential for DIW 3D printing applications, as it enables ink extrusion through the nozzle under relatively low pressure conditions.^[^ ^33^ ^]^ When subjected to higher shear rates up to 100 s^−1^, both inks exhibited a significant viscosity reduction to 740.8±134.3Pa∙s, demonstrating their favorable response to extrusion forces and ensuring smooth flow through the printing nozzle.

The shear‐thinning behavior plays a critical role in determining the ink's printability, while the ink's modulus is equally essential for maintaining shape fidelity of the printed structures. As demonstrated in Figure 1d, The quantitative data indicate that at a shear stress of 10 Pa, the G′ of the CP ink is 5477.5 ± 7.07 Pa and the G″ is 669.97 ± 2.19 Pa. After modification, the G′ and G″ of the CP‐SL‐Fe ink increase to 8317.5 ± 62.91 and 1039.05 ± 28.63 Pa, respectively. Both inks displayed solid‐like characteristics, evidenced by their storage modulus (G′) exceeding the loss modulus (G″) by more than eightfold.^[^ ^34^ ^]^ Notably, the modified CP‐SL‐Fe ink showed enhanced mechanical properties, with both storage modulus (G′) and loss modulus (G″) significantly higher than those of the unmodified CP ink, confirming the effectiveness of the modification process. The crossover point of the G′ and G″ curves indicated that the yield stress (τy) of CP ink was ≈237 Pa, while the τy of CP‐SL‐Fe ink was 194 Pa. Although crosslinking typically increases stiffness, the observed decrease in yield stress for CP‐SL‐Fe compared with CP can be attributed to microstructural and physicochemical effects arising from the mild ionic crosslinking process: i) The unmodified CP ink relies mainly on physical chain entanglements, whereas CP‐SL‐Fe contains localized Fe^3+^–ligand coordination points. Under shear, these ionic crosslinks may undergo concentrated, reversible rupture once a critical stress is reached, leading to yielding and flow initiation at a lower macroscopic stress level—analogous to a stress relaxation mechanism. ii) The incorporation of metal salts introduces trace amounts of water, which act as an internal plasticizer, weakening intermolecular interactions and increasing chain mobility.^[^ ^30^, ^34^, ^35^ ^]^ This effect further facilitates deformation and reduces the apparent yield stress. iii) Importantly, this controlled reduction in yield stress is beneficial for printing performance, as it allows smoother extrusion under lower pressure. After extrusion, the rapid reformation of ionic crosslinks enables quick modulus recovery, maintaining structural fidelity during layer deposition.

Overall, these results suggest that while Fe^3+^ crosslinking enhances the viscoelastic strength of the ink (evidenced by higher G′ and G″), the local reversibility and hydration effects of ionic interactions lead to a moderate decrease in yield stress—a trade‐off that optimizes extrusion flow characteristics and improves processability without compromising structural integrity. After extrusion, the modulus recovers rapidly due to the reformation of ionic bonds, which in turn optimizes printability. Importantly, both ink formulations exhibited smooth extrusion and good printability throughout the printing process.

### 2.3 Analysis of Surface Morphology and Structure

**Figure 2:**
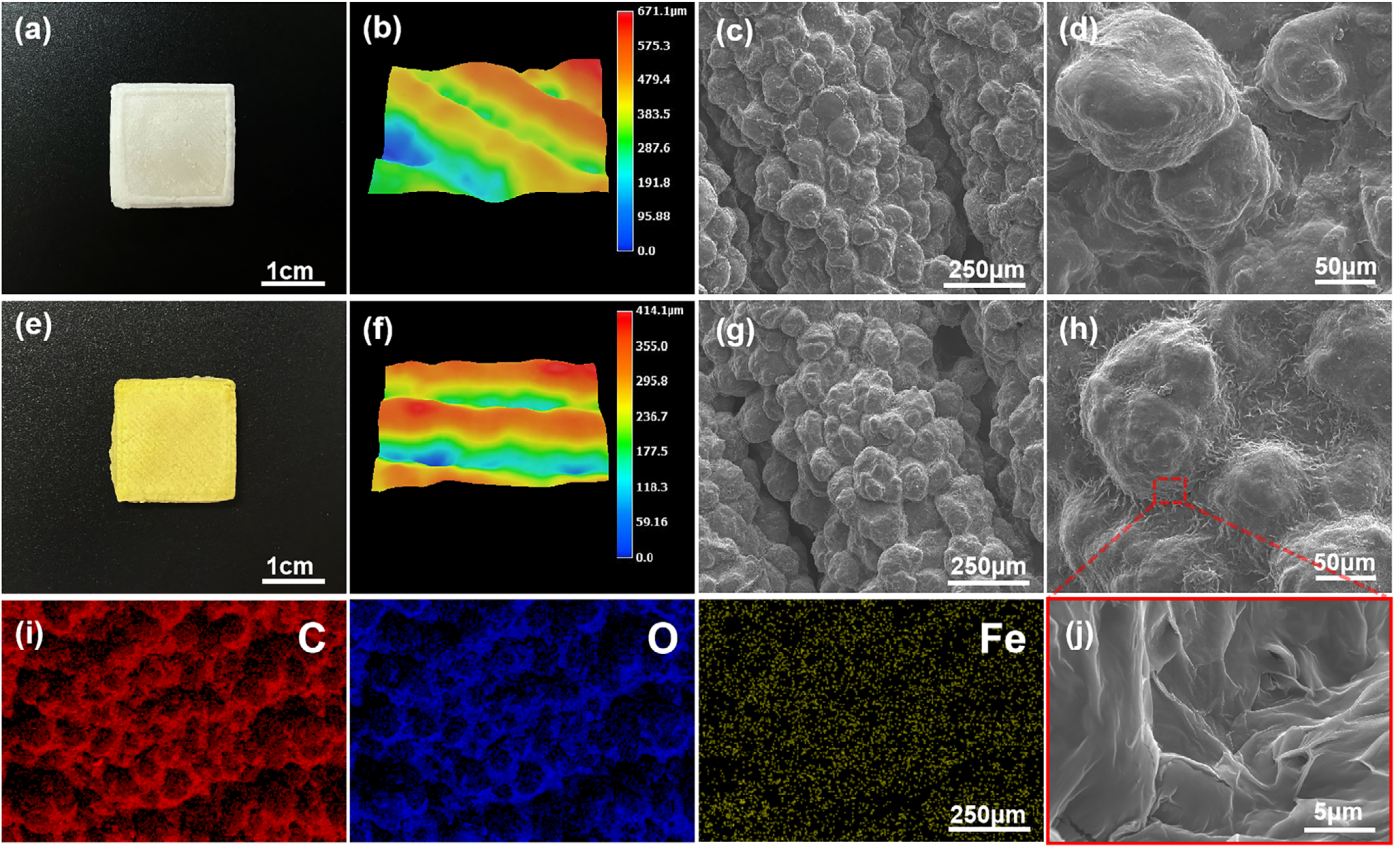
Morphological characterization of CP and CP‐SL‐Fe matrices. a,e) Photographs of sample appearance. b,f) 3D‐SDF Microscope images. c,d,g,h,j) SEM images. i) EDS mapping spectra of CP‐SL‐Fe.

3D‐printed carriers must satisfy multiple requirements for green agriculture applications, while maintaining structural stability during transportation and field application under complex environmental conditions. In this study, we successfully printed pheromone‐loaded matrices (CP and CP‐SL‐Fe) using both ink formulations (**Figure** **2**a,e). The white‐colored CP turns yellow upon SLS/Fe^3+^ incorporation to form CP‐SL‐Fe, demonstrating a visible color transformation. The surface morphology of the printed matrices was thoroughly characterized using 3D Super Depth‐of‐Field Microscope (Figure 2b,f). Microscopic analysis revealed that both the printed carriers displayed well‐defined surface patterns with uniform elevation‐depression profiles, demonstrating print integrity without detectable structural defects.

The surface morphology of 3D‐printed samples was characterized using scanning electron microscopy (SEM). As shown in Figure 2c,g, CA formed distinct agglomerated binding structures under the combined influence of organic solvents and PEG in CP and CP‐SL‐Fe, indicating that the ink underwent intensive crosslinking under shear forces during the printing process. While the CP displayed a relatively smooth surface (Figure 2d), the CP‐SL‐Fe exhibited a characteristic wrinkled morphology (Figure 2h,j). This structural transformation can be attributed to the coordination crosslinking effect between Fe^3+^ and polymeric components (CA, PEG, and SLS), aligning with previous research findings.^[^ ^36^, ^37^ ^]^ Furthermore, elemental distribution analysis through energy‐dispersive spectroscopy (EDS) confirmed the homogeneous dispersion of Fe^3+^ ions throughout the CP‐SL‐Fe carrier (Figure 2i). To further quantify these morphological changes, surface analysis was performed using Atomic Force Microscopy (AFM). As summarized in Table S1 (Supporting Information), the roughness parameters of CP‐SL‐Fe (Rq: 57.1 nm; Ra: 45.6 nm) were significantly higher than those of CP (Rq: 32.0 nm; Ra: 24.6 nm), indicating a more pronounced surface topography. The AFM 3D topography images (Figure S1, Supporting Information) visually revealed more wrinkles and cluster‐like structures on CP‐SL‐Fe, with a Z‐range variation (±350.0 nm) approximately double that of CP (±180.0 nm), further confirming the surface structural changes induced by crosslinking. These results collectively demonstrate successful material modification with uniformity and structural stability.

### 2.4 Investigation of Chemical Structure and Properties

**Figure 3:**
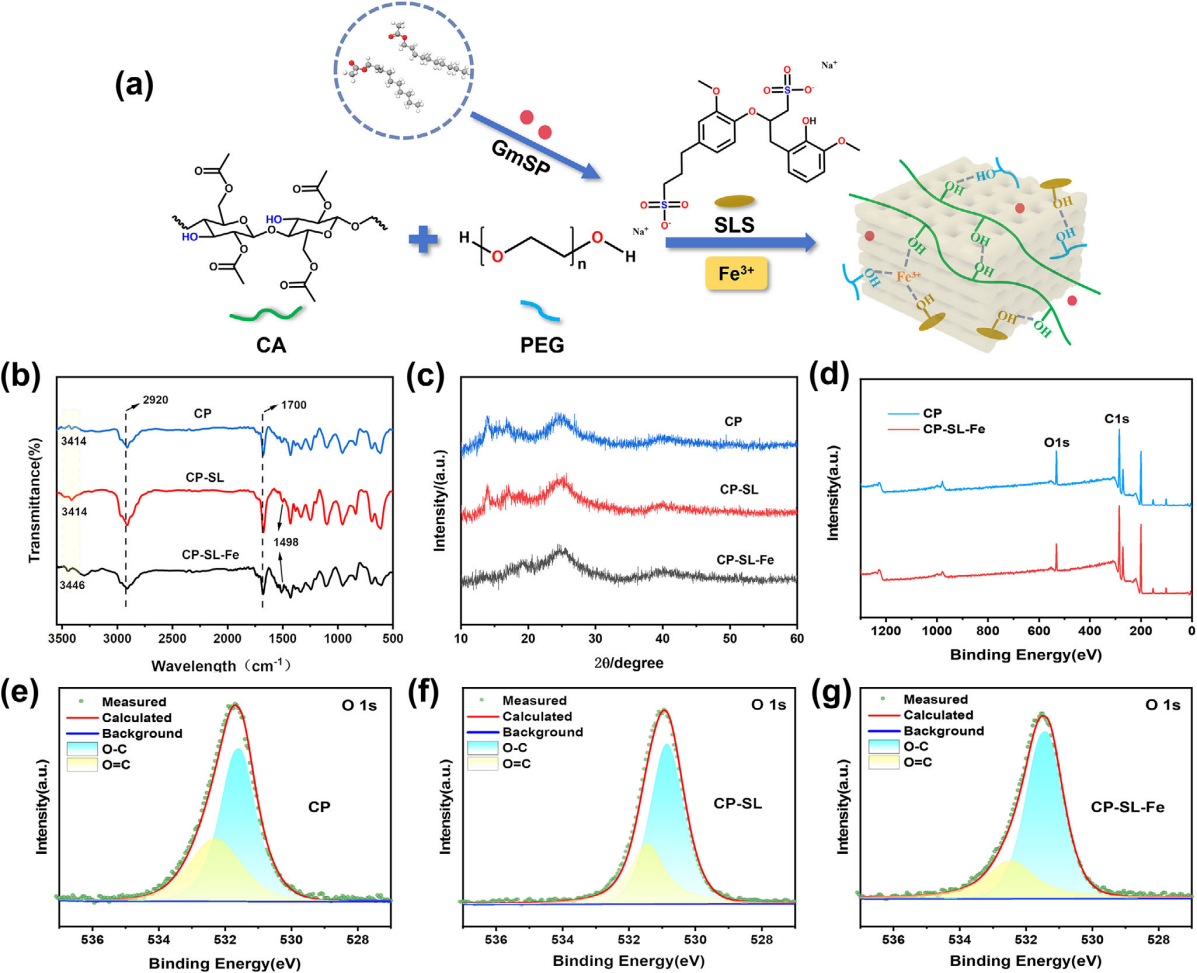
a) Schematic representation of the interaction of polymers (CA, PEG, SLS) with Fe^3+^ and pheromones. b) FTIR spectra. c) XRD patterns. d) XPS scans. e) O1s XPS spectra of CP. f) O1s XPS spectra of CP‐SL. g) O1s XPS spectra of CP‐SL‐Fe.

Fourier‐transform infrared spectroscopy (FTIR) analysis was conducted to characterize the chemical structures of 3D‐printed pheromone carriers, including CP, CP‐SL (the intermediate product with SLS but without Fe^3+^), and CP‐SL‐Fe, as shown in **Figure** **3**b. The absorption band at 1700 cm^−1^ was associated with acetate groups (C═O stretching), while the peak at 2920 cm^−1^ represented symmetric and asymmetric C‐H stretching vibrations of methyl groups.^[^ ^38^ ^]^ Comparing the spectra of CP and CP‐SL‐Fe, a shift of the absorption peak near 1250 cm^−1^, attributed to C─O stretching vibration, to a higher wavenumber was observed, accompanied by corresponding changes in the C═O absorption peak. This systematic peak shift is attributed to the coordination between Fe^3+^ and functional groups such as hydroxyl (─OH) and carboxylate (─COO^−^) on the polymer chains. The strong electron‐withdrawing effect of Fe^3+^ reduces the electron cloud density on the oxygen atoms, thereby increasing the force constant of the C─O bond, leading to the observed blue shift, and concurrently affecting the electron distribution of the C═O group.^[^ ^39^ ^]^ After the introduction of SLS, an absorption peak at 1498 cm^−1^ appeared, suggesting the presence of aromatic ring vibrations in both CP‐SL and CP‐SL‐Fe.^[^ ^40^ ^]^ Comparative analysis of CP, CP‐SL and CP‐SL‐Fe spectra revealed a significant shift in the −OH stretching vibration from 3414 in CP to 3446 cm^−1^ in CP‐SL‐Fe. This spectral shift suggested that Fe^3+^ modification induced coordination bonding with hydroxyl groups, consequently weakening intermolecular hydrogen bonding.^[^ ^35^, ^41^ ^]^ The above phenomena preliminarily demonstrated that the CP‐SL‐Fe carrier formed a more complex cross‐linked coordination structure through hydrogen bonds and coordination bonds, as illustrated in Figure 3a.

X‐ray diffraction (XRD) analysis was employed to investigate crystallographic changes before and after modifications. As shown in Figure 3c, the CP and CP‐SL carriers displayed characteristic diffraction peaks of cellulose I at 2θ = 14.1° and 24.5°. The characteristic peak at 2θ = 14.1° disappeared in the CP‐SL‐Fe carriers, suggesting that the introduction of metal ions changed intermolecular interactions within the composite material, consequently reducing its crystallinity.^[^ ^42^, ^43^ ^]^ X‐ray photoelectron spectroscopy (XPS) was utilized to monitor chemical structure evolution. Compared to the CP and CP‐SL carrier, the XPS data of the CP‐SL‐Fe carrier (Figure 3e,f,g) showed a significant increase in the content of C─O groups, while the content of C═O groups slightly decreased. These changes can be attributed to the incorporation of SLS, which introduces additional hydroxyl and ether groups. Meanwhile, Fe^3+^ may coordinate with the oxygen‐containing functional groups (such as hydroxyl and ether groups) in the SLS molecules, thereby enhancing the crosslinking effect. The Fe^3+^ coordination not only improves the material's stability but may also change the distribution and properties of surface functional groups.^[^ ^44^, ^45^, ^46^ ^]^ Taken together, the changes observed in FTIR and XPS spectra, along with the reduction in crystallinity revealed by XRD, collectively support the successful formation of a coordinatively crosslinked structure following Fe^3+^ introduction.

### 2.5 Physical Properties and Degradation Behavior

**Figure 4:**
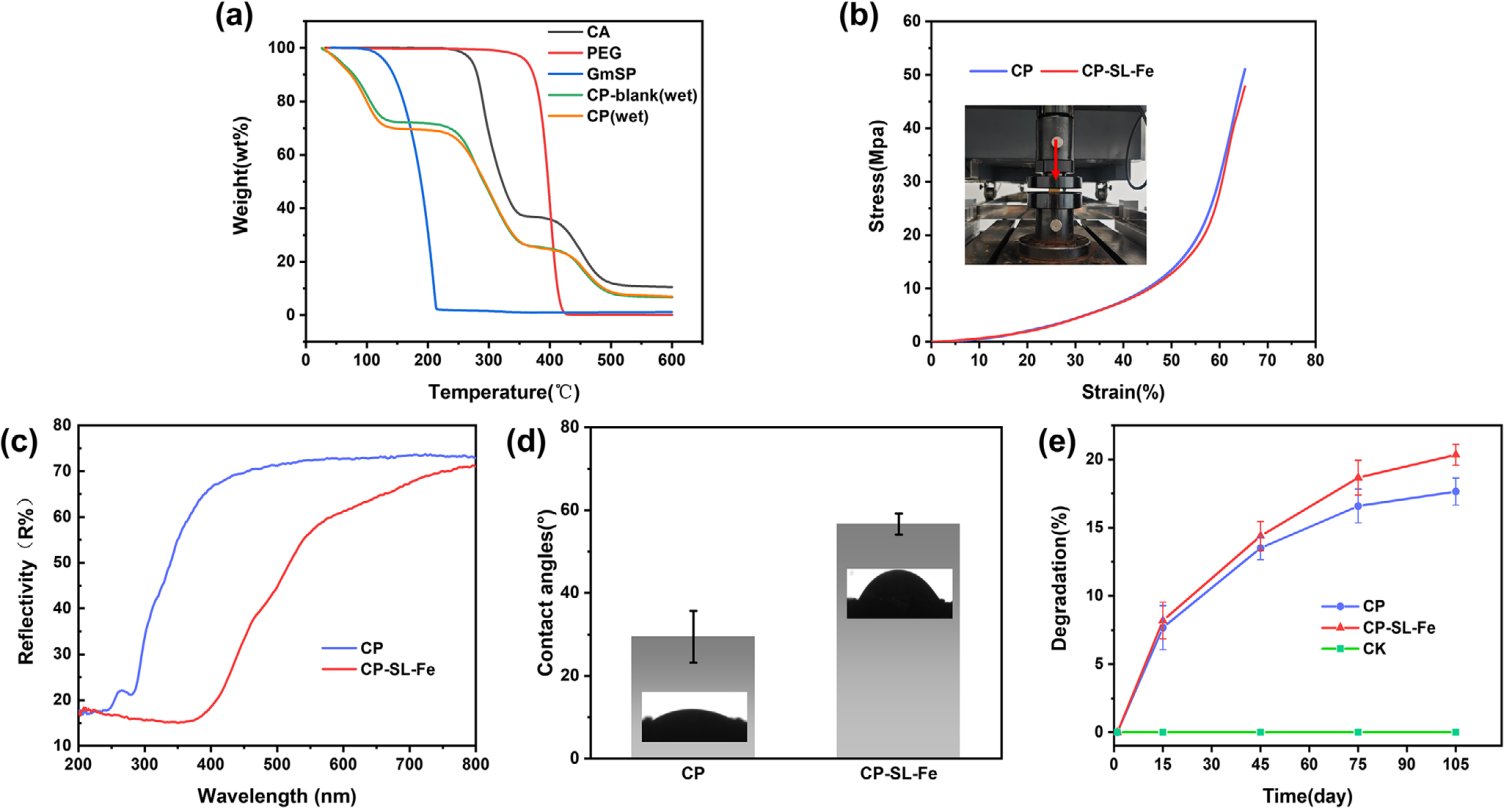
a) TGA thermal weight loss curve. b) Stress‐strain curve. c) UV‐visible diffuse reflectance. d) Water contact angle measurement. e) Comparison of soil degradation weight loss rates between 3D‐printed carriers and commercial rubber carriers.

Thermogravimetric analysis (TGA) was performed to investigate the thermal degradation behavior of 3D‐printed samples, in order to systematically analyze all components of the printed carrier material, including possible residual solvents, PEG, pheromones, and cellulose. For this purpose, the samples used in this test were undried (labeled as “wet”). The samples included CP‐blank (empty carrier), CP, CA, PEG and GmSP, over a temperature range of 25–600 °C (**Figure** **4**a). Both CP‐blank(wet) and CP(wet) exhibited a mass loss of ≈25% within the temperature range of 40–150 °C, which is primarily attributed to the removal of residual acetone and DMF solvents. Furthermore, thermogravimetric analysis (TGA) was performed on the dried sample (CP) and compared with CP‐wet (Figure S2, Supporting Information). The results showed no mass‐loss plateau for CP between 40 and 150 °C, confirming that the drying process effectively removed the residual solvents. Given that residual high‐boiling‐point DMF may pose a risk to the safe use of the material, gas chromatography (GC) was employed to quantitatively analyze the DMF content in CP samples before and after drying, so as to further evaluate the safety of the material. As presented in Table S2 (Supporting Information), the DMF content in the CP sample was extremely low (0.27 ± 0.05 wt.%), whereas the CP‐wet sample showed a significantly higher DMF content (27.34 ± 1.32 wt.%). These results collectively demonstrate a significant reduction of DMF to a very low level in the dried CP samples, suggesting a favorable safety profile for practical applications.

**Figure 5:**
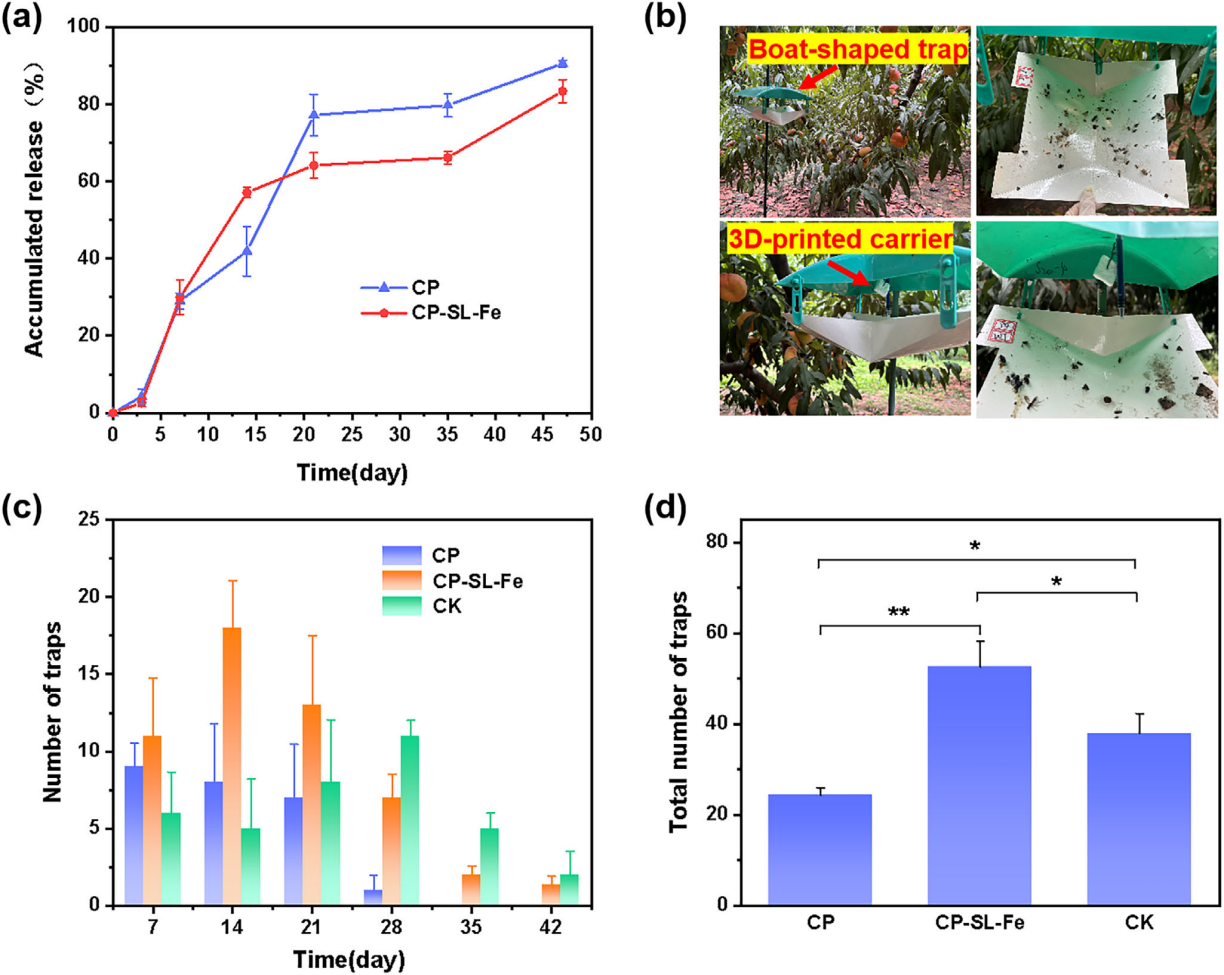
a) Pheromone release curves in the laboratory. b) Photographs of the field experiment. c) Weekly trapping amount in the field. d) Cumulative trapping amount in the field (6 weeks). Total number of *G. molesta* entrapment of different pheromone carriers (6 weeks). Three samples were fabricated and tested for each group. A One‐Way ANOVA followed by a Tukey‐Kramer honestly significant difference (HSD) test was used to compare means of all treatments. Asterisks denote the level of significance: *p‐value < 0.05, **p‐value < 0.01, and ***p‐value < 0.001.

The decomposition of GmSP initiates at 90 °C and is fully volatilized by 215 °C. Over the same temperature range, the CP sample exhibits a 3% higher weight loss rate compared to the CP‐blank, indicating successful loading of GmSP onto the carrier. For the carrier material, thermal weight losses of 43% and 14% observed between 250–350 and 400–500 °C, respectively, correspond to the deacetylation/volatilization of CA and the decomposition of the polymer backbone.^[^ ^47^ ^]^ The weight loss observed between 350 and 400 °C can be attributed to the cleavage and decomposition of PEG. The release kinetics of the encapsulated pheromone are strongly temperature‐dependent, as supported by the TGA results. The pure pheromone (GmSP) began to volatilize significantly at ≈90 °C (Figure 4a), reflecting a rapid increase in vapor pressure and diffusion rate within this range. However, when encapsulated within the CA‐based carrier, particularly the Fe^3+^‐coordinated CP‐SL‐Fe network, pheromone volatilization below 90 °C was substantially suppressed (**Figure** **5**a). This behavior indicates that the carrier transforms the temperature‐driven volatilization process into a diffusion‐limited release governed by molecular interactions within the polymer matrix. The observed slower release rate of CP‐SL‐Fe compared with CP at 25 °C further supports this mechanism. In thermodynamic terms, the Fe^3+^ coordination and hydrogen bonding interactions likely increase the apparent activation energy of diffusion, thereby moderating the influence of temperature on the release rate. Consequently, the carrier not only encapsulates the pheromone but also actively regulates its temperature‐dependent diffusion, ensuring stable and prolonged release performance under fluctuating field conditions.

The compression test results revealed that the CP carriers exhibited a compressive strength of 51.06 MPa and a compressive modulus of 1.47 GPa. In contrast, the CP‐SL‐Fe demonstrated significantly enhanced properties, with a compressive strength of 87.96 MPa and a compressive modulus of 1.87 GPa. These mechanical property data confirm that the fabricated printing carrier possesses sufficient strength to withstand the demands of transportation and application.^[^ ^48^ ^]^ The superior compressive strength of the CP‐SL‐Fe compared to CP suggested its enhanced resistance to deformation, indicating higher structural stability. Furthermore, analysis of the stress‐strain curves at 65% strain (Figure 4b) showed similar trends for both materials, indicating consistent fundamental deformation mechanisms. Additionally, we have supplemented the study with tensile property tests. As shown in Figure S3a (Supporting Information), dog bone‐shaped specimens were prepared and tensile tests were conducted using a universal testing machine. The resulting stress‐strain curves in Figure S3b (Supporting Information) indicate that the CP material exhibited higher tensile strength (3.96 MPa) and modulus (85.14 MPa), but a lower fracture strain (80.27%), demonstrating brittle characteristics. In contrast, CP‐SL‐Fe showed a decrease in tensile strength (2.02 MPa) and modulus (4.34 MPa), while the fracture strain increased to 117.89%, indicating a certain degree of toughening or a transition toward more ductile behavior. This brittle‐to‐ductile shift may be related to the metal ion crosslinking and the associated internal plasticizing effect due to the introduction of water molecules, which weaken intermolecular interactions and enhance chain segment mobility.^[^ ^35^, ^49^, ^50^ ^]^ The material exhibited different response mechanisms under compressive and tensile loads: under compression, metal ion crosslinking helps inhibit molecular chain buckling, thereby enhancing compressive resistance. However, during tension, the layered structure inherent to the 3D‐printed samples might expose interlayer weaknesses more readily. The improvement in interlayer bonding strength after modification could also be a significant factor contributing to the enhanced compressive performance.^[^ ^34^, ^51^ ^]^

Ultraviolet‐visible‐near infrared diffuse reflectance spectroscopy (Figure 4c) revealed that CP exhibited significantly higher reflectance in both the ultraviolet and visible light regions compared to CP‐SL‐Fe. This difference can be attributed to the inherent light absorption properties of SLS, whose incorporation enhances the absorption capacity of samples, particularly in the ultraviolet band.^[^ ^52^ ^]^ Ultraviolet protection is critically important in the design of insect pheromone delivery systems, as most pheromone molecules contain chemical bonds susceptible to UV degradation, such as ester linkages, aldehyde groups, or unsaturated carbon‐carbon double bonds. Prolonged exposure to ultraviolet radiation in sunlight can induce photolysis or photo‐oxidation, leading to pheromone deactivation and reduced trapping efficiency.^[^ ^53^ ^]^ The decreased reflectance (indicating enhanced absorption) of CP‐SL‐Fe is due to the introduced SLS. As a natural aromatic polymer, SLS contains conjugated systems (e.g., benzene rings) that confer strong ultraviolet absorption capabilities.^[^ ^29^, ^54^ ^]^ Therefore, SLS can function as a UV protectant for pheromones to some extent, acting as a built‐in “UV shield” within the polymer matrix to absorb and dissipate UV energy, thereby slowing the photodegradation of encapsulated pheromone and helping maintain its long‐term bioactivity under field conditions. Furthermore, water contact analysis demonstrated a remarkable increase in water contact angle after modification (Figure 4d). The water contact angle increased significantly from 30.2±5.4° for CP to 56.6±2.5° for CP‐SL‐Fe. Although the increase indicates reduced surface polarity, the obtained value still corresponds to a hydrophilic surface, rather than a hydrophobic one. This moderate change can be attributed to the coordination interactions between Fe^3+^ ions and polar functional groups (e.g., hydroxyl and sulfonate groups) within the CA–PEG–SLS polymer matrix, which partially shielded hydrophilic moieties and reduced their exposure on the surface. However, the relatively low Fe^3+^ concentration used in this study was insufficient to fundamentally alter the intrinsic hydrophilic nature of the cellulose‐based material. While the surface did not become hydrophobic, this moderate reduction in wettability is still beneficial for field durability. The increased contact angle helps to alleviate the immediate effects of environmental moisture (such as dew or light rainfall) on the carrier surface, thereby helping to maintain the structural integrity and release stability of the pheromone‐loaded material under outdoor conditions.

Given that most materials currently used for pheromone loading (rubber, polyethylene, etc.) are petroleum‐based and non‐biodegradable, we chose CA, which is more easily degradable, as the carrier matrix. The degradation behavior of 3D‐printed samples was evaluated under natural soil conditions, as illustrated in Figure S4 (Supporting Information). The weight loss curves (Figure 4e) showed a progressive increase in mass loss over time for the 3D‐printed samples, whereas the commercial rubber samples (CK) maintained nearly constant throughout the experimental period. After 105 days of soil burial, the CP and CP‐SL‐Fe exhibited weight loss rates of 17.7±1.0% and 20.4±0.8%, respectively. Therefore, it can be preliminarily concluded that the samples undergo slow degradation. The primary microorganisms responsible for degrading the 3D‐printed carriers were fungi, bacteria, and actinomycetes, which are known decomposers of cellulose and lignin structures.^[^ ^55^, ^56^ ^]^ Although the soil burial results indicated a relatively slow degradation rate of the carrier, consistent with the gradual biodegradation behavior typical of CA materials.^[^ ^57^, ^58^ ^]^ This stability ensures that the carrier maintains its functional integrity during the effective field period (>6 weeks). Based on the observed trend, complete degradation is estimated to occur over a timescale exceeding one year, which, although not rapid, still represents a significant improvement in environmental sustainability compared with non‐degradable rubber or plastic carriers. The Fe^3+^‐induced coordination network likely contributes to this slow initial degradation by enhancing structural stability and reducing water penetration, while the presence of SLS and Fe^3+^ may later promote degradation through microbial utilization of lignin‐derived carbon sources and Fe^3+^‐catalyzed oxidative processes.^[^ ^59^, ^60^ ^]^ Together, these factors suggest that the crosslinked carrier exhibits degradability.

### 2.6 Pheromone Encapsulation and Release Profiles

Pheromone encapsulation efficiency and release kinetics represent crucial aspects in the development of carrier delivery systems. The experimental results showed that the encapsulation efficiencies of the CP and CP‐SL‐Fe carriers were 95.4 ± 4.1% and 96.2 ± 2.7%, respectively (Figure S5, Supporting Information), indicating that the DIW process did not result in significant pheromone loss and was therefore feasible. The release profiles of GmSP from both carrier systems were presented in **Figure** **5**a. Both 3D‐printed samples exhibited two‐stage release profile, consisting of an initial burst release followed by sustained release over four weeks. During the first two weeks, GmSP was rapidly released from the carrier surface. By the second week, cumulative release rates reached 41.9 ± 6.4% for CP and 57.2 ± 1.3% for CP‐SL‐Fe. Previous studies have proposed that the initial burst release originates from the surface and amorphous regions of the carriers, while sustained release primarily occurs from crystalline domains.^[^ ^61^ ^]^ After two weeks, CP‐SL‐Fe demonstrated a slower release rate compared to CP. By day 47, cumulative release rates reached 83.4 ± 3.0% for CP‐SL‐Fe and 90.6 ± 0.9% for CP. This modified release behavior can be attributed to enhanced intermolecular interactions, including coordination bonds and hydrogen bonding, within the polymeric network of CP‐SL‐Fe. These interactions promoted the formation of a more compact matrix structure, effectively encapsulating the pheromone within the crosslinked network and thereby modulating the release rate.^[^ ^62^, ^63^ ^]^

To comprehensively investigate the pheromone release kinetics from 3D‐printed carriers, we employed four widely‐used drug release models for simulation analysis: Zero‐order, First‐order, Higuchi, and Ritger–Peppas models (Table S3, Supporting Information). Comparative analysis of the model fitting results revealed that both CP and CP‐SL‐Fe carrier systems showed the best correlation with the first‐order kinetic model, as evidenced by their highest R^2^ values (0.984 and 0.873). This finding suggested that the pheromone release followed first‐order kinetics, where the release rate was proportional to the remaining drug concentration. Preliminary laboratory release studies indicate that the 3D‐printed pheromone delivery systems hold promising potential for achieving sustained pest control efficacy under field conditions.

In summary, these results indicate that Fe^3+^‐induced coordination and hydrogen bonding effectively strengthened the polymer network, reducing molecular mobility and achieving a more sustained pheromone release. This crosslinking‐driven diffusion control provides a key functional advantage for enhancing the long‐term stability and efficacy of the delivery system.

### 2.7 Field Trapping Performance for *G. molesta*

The application performance and biological activity of pheromone carriers under field conditions were evaluated through field experiments. Figure 5b illustrated the practical implementation and trapping of 3D‐printed pheromone samples in boat‐shaped traps under field conditions. Analysis of the six‐week trapping data (Figure 5c) revealed superior trapping efficacy of the 3D‐printed carriers, with CP‐SL‐Fe demonstrating better trapping performance than CP and comparable longevity to CK. Compared to CK, CP‐SL‐Fe exhibited higher trapping numbers during the first three weeks, consistent with its characteristic of rapid release in the early stages. The six‐week cumulative trapping data (Figure 5d) showed that CP‐SL‐Fe reached a catch of 53 ± 6, significantly higher than that of CP (24 ± 2) and the commercial rubber carrier (CK, 38 ± 4) (*p* = 0.0012 and 0.0294, respectively), demonstrating its stronger field trapping capability for G. molesta. These field results provided compelling evidence for the effectiveness of 3D‐printed pheromone carriers in complex environmental conditions. The long‐term and better trapping performance of CP‐SL‐Fe can be attributed to both the cellulose‐based composites themselves and their optimized internal chemical crosslinking, which together enable the protection of pheromones and regulation of their release behavior. Although field trials have confirmed the practical effectiveness of 3D‐printed carriers in the control of *Grapholita molesta*, the uniformity of the sustained release during the mid‐to‐late stages of the current carriers still requires optimization. It is important to emphasize that 3D printing technology itself offers unique tunable advantages in this regard: on one hand, the formulation of functional inks can be precisely adjusted at the molecular level (e.g., by modulating coordination bond strength) to finely regulate release kinetics; on the other hand, the macroscopic design of the printed structure (such as the interlayer architecture) enables the programming of multiple release channels. This integrated material–structure control capability is difficult to achieve with traditional rubber carriers prepared by solvent permeation methods. Although the current mid‐term release stability has not yet reached the optimum, the higher cumulative trapping efficacy and comparable duration of effectiveness have demonstrated the practical applicability of this carrier. These findings establish a strong foundation for future research on sustained‐release 3D‐printed carrier systems and their practical applications in pest management. With further optimization of material formulations and printing technology, the 3D‐printed pheromone delivery system is expected to enhance sustained release effects and resistance to complex environments.

In summary, the significant advantages of the CP‐SL‐Fe carrier in terms of mechanical strength, sustained pheromone release performance, and field trapping efficacy can be attributed to the three‐dimensional crosslinked network constructed via 3D printing technology and the cellulose‐based composite material. This network acts as “molecular anchors,” restricting polymer chain slippage and thereby increasing the compressive strength from 51.06 to 87.96 MPa, which enhances the structural stability of the carrier. Concurrently, although the crosslinking‐induced matrix densification, accompanied by reduced crystallinity, extends the diffusion path for the pheromone, the stronger coordination bonds and hydrogen bonding interactions introduce significant diffusion resistance. This results in a more gradual release profile after the initial burst, lowering the cumulative release rate to 83.4 ± 3.0% over 47 days, with the release behavior fitting a first‐order kinetic model. Ultimately, the synergistic effect of the reinforced mechanical framework and the controlled diffusion barrier enables the CP‐SL‐Fe carrier to exhibit both high trapping efficiency and long‐lasting efficacy in the field. This successfully establishes a coherent link from the microscopic molecular structure to the macroscopic application performance.

### 2.8 Exploration of Carrier Recyclability

**Figure 6:**
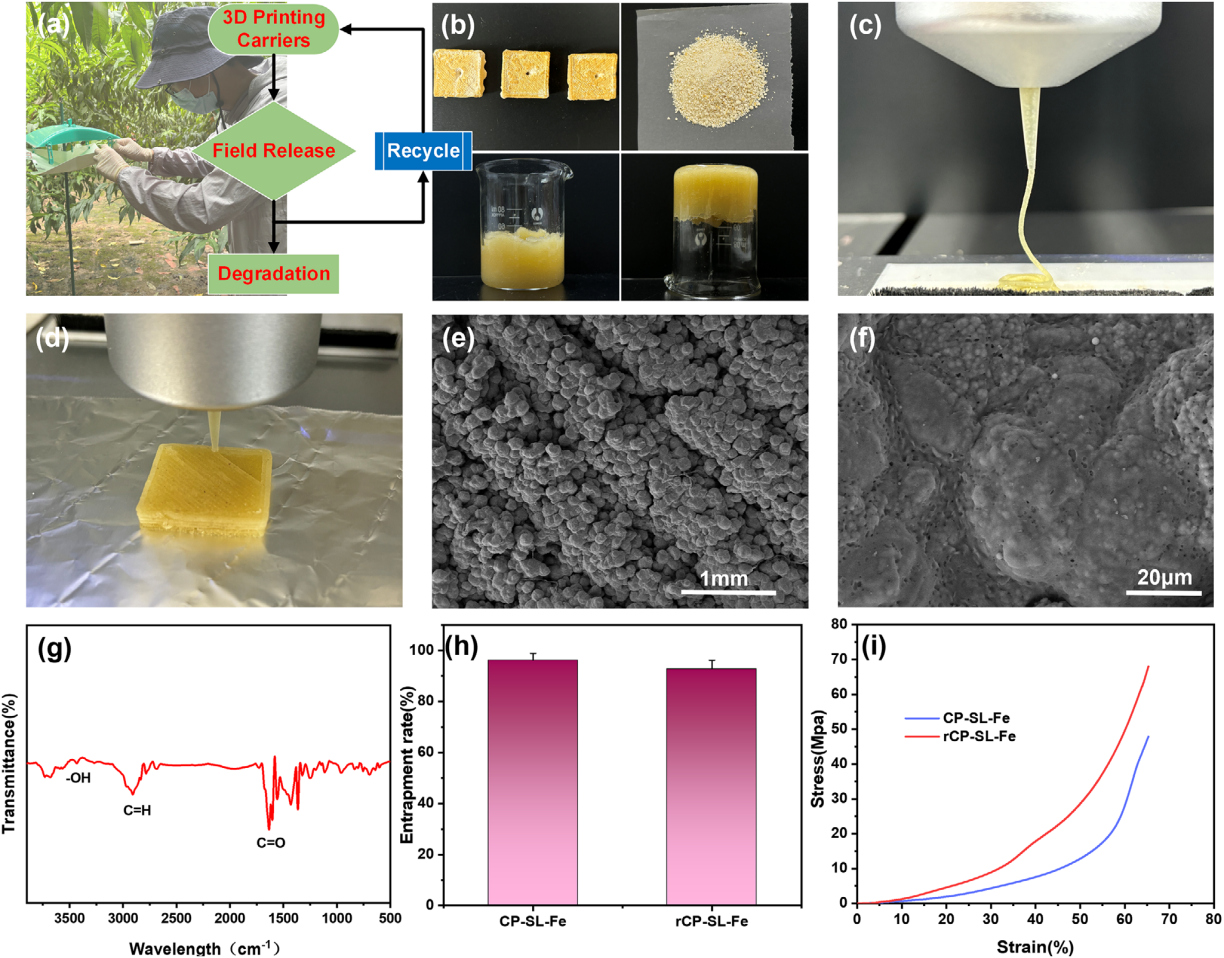
a) Roadmap of green treatment. b) Photographs of recycling treatment experiments (recycled carriers; physical grinding; ink in both upright and inverted glassware). c,d) Photographs of recycled ink printing smoothness and plasticity shape. e,f) SEM morphology of rCP‐SL‐Fe. g) FTIR spectra of rCP‐SL‐Fe. h) Encapsulation rate comparison between CP‐SL‐Fe and rCP‐SL‐Fe carriers. i) Stress–strain comparison between CP‐SL‐Fe and rCP‐SL‐Fe carriers.

According to current literature, neither the widely used commercial products nor the new carrier materials under development have systematically reported on their recyclability and reuse. Therefore, we recycled and reprinted the carriers after long‐term use in the field, and then evaluated their physical and chemical properties as well as encapsulation performance. The recycling process, illustrated in **Figure** **6**a,b, involved the recovery of field‐collected carriers followed by ink formulation through physical grinding, organic solvent dissolution and pheromone supplementation. Remarkably, the recycled ink retained excellent extrusion and molding properties, as shown in Figure 6c,d. Through the simple reprocessing experiments described above, we successfully recycled and reutilized the CP‐SL‐Fe carriers recovered from the field and fabricated re‐printable pheromone carriers (rCP‐SL‐Fe). Microstructural characterization through SEM‐EDS analysis revealed that the reprinted carrier displayed a densely crosslinked structure (Figure 6e,f), with well‐organized morphology and homogeneous iron distribution (Figure S6, Supporting Information), preserving surface morphology characteristics similar to CP‐SL‐Fe. FTIR spectroscopy results (Figure 6g) showed absorption peaks at 1643 and 2907 cm^−1^, which correspond to the C═O stretching vibration of the acetate group and the symmetric and asymmetric C─H stretching vibrations of the methyl group. Additionally, a stretching vibration peak at 3431 cm^−1^ is observed for the ‐OH group, further confirming the chemical consistency with previous results. As noted, the CP‐SL‐Fe sample was recovered after six weeks of real‐field use and subjected to a second printing cycle. During this period, the material surface may have been slightly contaminated by environmental factors, which could account for the observed variations in the FTIR spectra. Nevertheless, the key characteristic absorption peaks consistent with the pristine CP‐SL‐Fe are still clearly identifiable in the spectra of the recycled material. In addition, XPS O 1s spectra of rCP‐SL‐Fe (Figure S7, Supporting Information) show no significant variation in the relative peak areas associated with C─O and C═O binding energies compared with the CP‐SL‐Fe, confirming that the chemical composition remained largely stable during recycling and reprinting. In summary, the combined evidence from FTIR, EDS, and XPS analyses consistently demonstrates that rCP‐SL‐Fe retains the same principal chemical structure as the original CP‐SL‐Fe. The recycled carrier achieved a high encapsulation efficiency of 92.8 ± 3.4%, indicating well‐preserved encapsulation capacity (Figure 6h). Moreover, the mechanical properties of the recycled material were enhanced, as evidenced by the stress‐strain curve (Figure 6i), with a compressive modulus of 124.28 MPa and a strength of 2.03 GPa. And the force‐displacement curve (Figure S8, Supporting Information) also demonstrated a significant improvement in the compressive performance of rCP‐SL‐Fe compared to CP and CP‐SL‐Fe. Additionally, the surface became less hydrophilic, with a water contact angle of 84.5 ± 8.1° (Figure S9, Supporting Information), exceeding the performance of the original CP‐SL‐Fe. These enhancements are attributed to the intensified crosslinking and structural optimization induced by the recycling process. Specifically, during recycling, the dissolution step partially breaks the original coordination and hydrogen bonds, enabling the polymer chains and additives (SLS and Fe^3+^) to redisperse more homogeneously within the matrix. Upon re‐printing and solidification, these components undergo reorganization and re‐coordination, allowing functional groups (e.g., ─OH) and Fe^3+^ ions to form additional and denser coordination bonds—a “secondary crosslinking” effect. This results in a more compact and mechanically reinforced network, consistent with the denser surface morphology observed in SEM images of rCP‐SL‐Fe (Figure 6e,f). Consequently, the improved network integrity significantly enhances the material's compressive strength. At the same time, this enhanced crosslinked structure reduces the mobility of surface hydrophilic groups and promotes the preferential orientation of hydrophobic moieties, contributing to the observed increase in water contact angle. In essence, the recycling process induces microstructural reorganization and network densification, which collectively strengthen the material and enhance its surface hydrophobicity.

To further assess recyclability, the rCP‐SL‐Fe material was reprocessed into printing ink and subjected to a third printing cycle to obtain r‐rCP‐SL‐Fe. The results (Figure S10, Supporting Information) show that the material maintained good printability and structural integrity through all three cycles, confirming the mechanical and morphological stability of the framework during repeated reprocessing. In order to validate the functionality of the recovered carriers, electroantennography (EAG) tests (Figure S11, Supporting Information) were employed to sensitively evaluate the biological activity of pheromones released from different recycling stages. Following the method of Zhong et al.,^[^ ^64^ ^]^ the stimuli for live male Grapholita molesta included CP‐SL‐Fe, rCP‐SL‐Fe, and r‐rCP‐SL‐Fe (each loaded with 0.5 mg of pheromone), with a blank carrier (CP‐SL‐Fe‐blank) as control. As shown in Figures S12,S13 (Supporting Information), the EAG response amplitudes for CP‐SL‐Fe, rCP‐SL‐Fe, and r‐rCP‐SL‐Fe all remained high, with no significant differences among the three groups. These results demonstrate that the pheromone release capacity and insect‐attracting efficacy of the carriers did not deteriorate even after multiple recycling and reprinting cycles.

In summary, encapsulation efficiency measurements, printability observations, and EAG bioassays collectively confirm that the CP‐SL‐Fe carrier maintains stable physical integrity and biological functionality after repeated recycling, demonstrating strong reusability and feasibility for sustainable field application—highlighting how the integration of simple processing methods with efficient 3D printing offers a promising approach to material recycling in pest management.

## 3 Conclusion

This study successfully developed biobased and efficient insect pheromone carriers using 3D printing technology, demonstrating significant potential for sustainable pest management. Pheromone‐loaded inks with optimized printability were formulated, yielding 3D‐printed samples with uniform surface morphology and stable physicochemical properties. Furthermore, the introduction of metal coordination chemistry enhanced the overall performance of the carrier, with CP‐SL‐Fe achieving over 95% encapsulation efficiency and demonstrating at least 6 weeks of indoor release and field trapping. These carriers exhibited a certain degradability, along with attractive recyclability and reuse properties, aligning well with the current demands for sustainable agricultural development. Overall, the DIW‐based 3D printing approach provides unique advantages over both conventional and other advanced fabrication methods. Its low‐temperature processing preserves pheromone activity, while its structural precision ensures uniform distribution and stable diffusion. In addition, the ability to recycle and reprint the carrier further strengthens its sustainability, establishing a scalable and eco‐efficient route for next‐generation pheromone delivery systems. These findings offer a viable pathway toward achieving a circular economy in agricultural pest control. As agricultural technologies evolve and interdisciplinary research advances, 3D printing stands out as a user‐friendly technology with significant potential for application and development in precision agriculture and sustainable farming practices.

## 4 Experimental Section

### Materials

*G. molesta* sex pheromone (GmSP) (mainly (Z, E)‐8‐dodecyl acetate), supplied by Pherobio Technology Co., Ltd (Beijing, China). Cellulose acetate (CA, *M*n = 60 000 g mol^−1^, acetyl content = 39.5 wt.%), Polyethylene glycol (PEG, *M*n = 4000 g mol^−1^), Sodium lignosulfonate (SLS), Anhydrous ferric chloride (FeCl_3_) were purchased from Macklin Biochemical Technology Co., Ltd (Shanghai, China). Analytically pure Acetone and *N, N*‐dimethylformamide (DMF) were obtained from Beijing Tong Guang Fine Chemicals Company. Analytical grade chemicals and reagent‐grade solvents were used in this study without further purification.

### Cellulose Acetate‐based Ink (CP ink) Preparation

At room temperature (25 °C), 2% (w/w) PEG was dissolved in a mixed solvent of acetone and DMF (4:1, v/v). Then, 0.2% of the *G. molesta* sex pheromone (pheromone mass to cellulose acetate mass ratio) was added and stirred at 500 rpm on a magnetic stirrer for 30 min until the solution became homogeneous. Next, 15% (w/w) CA was added and mechanically stirred at 800 rpm for 5 min, resulting in a semi‐solid printable ink. To remove air bubbles, the resulting ink was sealed in a closed container and allowed to stand for 12 h. The ink can be stored in a sealed container at 4 °C and remains usable for up to one week.

### Modified Cellulose Acetate‐based Ink (CP‐SL‐Fe ink) Preparation

SLS and FeCl_3_ were prepared into an aqueous solution with a concentration of 0.5 g mL^−1^. Following the previously described CP ink preparation steps, 0.1% (w/w) SLS and 0.5% (w/w) FeCl_3_ were gradually added after the complete dissolution of PEG. The remaining steps were conducted in the same manner as the CP ink preparation.

### 4.1 Printing of CP and CP‐SL‐Fe

This study uses a cubic model for printing, with dimensions of 20 × 20 × 10 mm. The modeling was performed using SOLIDWORKS software, and slicing was done using Repetier‐Host software. The cellulose‐based ink containing mixed pheromones was printed using an extrusion‐style 3D printer (Foodbot‐D1, Shiyin Technology). The printing parameters are as follows: nozzle diameter of 0.8 mm, print layer height of 1 mm, with the first layer having a height of 0.8 mm; fill density set at 90%; and print speed of 15 mm s^−1^. The printing parameters for both inks are identical, and both were extruded and printed at room temperature (25 °C). Additionally, the printing environment was well‐ventilated. To facilitate rapid shaping, all samples were dried at 60 °C for 10 h to remove residual solvents.

### Measurement of Rheological Properties

This study analyzes the rheological properties of the ink using a rheometer (MCR502, Anton Paar, Austria). The rheological performance tests include shear viscosity tests and oscillatory stress sweep tests, both conducted using a PP25 parallel plate (diameter 25 mm, gap 1 mm). The tests were performed under constant temperature conditions at 25 °C. Shear viscosity analysis was conducted over a shear rate range of 0.1 to 100 s^−1^. The oscillatory stress sweep analysis was performed under a constant frequency of 10 rad s^−1^ and a strain range of 1% to 1000%, from which the ink's storage modulus (G′) and loss modulus (G″) were obtained.

### Characterization of Surface Morphology

The surface morphology of the 3D‐printed matrices were obtained using a scanning electron microscope (SEM, SU8010). Before observation, a 9 nm platinum coating was applied to the surface of the substrate, and the working voltage of the machine was set to 10 kV. The surface topographical variations of the substrate were observed using a 3D super depth‐of‐field microscope (VHX‐200). The surface roughness of samples CP and CP‐SL‐Fe was characterized using an atomic force microscope (AFM, Bruker Dimension Icon, Germany). Different areas of the samples were scanned, and their arithmetic average roughness (Ra) and root mean square roughness (Rq) were calculated for quantitative analysis.

### Chemistry Characterization

This study employs various characterization techniques to analyze the chemical and crystalline structures of 3D‐printed carriers before and after modification. Attenuated Total Reflectance Fourier Transform Infrared Spectroscopy (ATR‐FTIR), Measured in ATR mode, with an instrument resolution set to 2 cm^−1^. The wavelength range is from 400 to 4000 cm^−1^, and the number of scans is set to 32. X‐ray Diffraction (XRD), using a copper target as the X‐ray source, the scanning angle range is set from 5° to 90°, with a scanning speed of 2° min^−1^ to obtain crystalline structure information of the carrier. X‐ray Photoelectron Spectroscopy (XPS), The passing energy during testing is set to 50 eV for full‐spectrum and 20 eV for narrow‐spectrum, with a step size of 0.05 eV and a dwell time of 40 to 50 ms. All test data are energy calibrated with the C1s peak (284.80 eV) as the reference, and charge correction is performed.

### Thermal Stability Characterization

Thermogravimetric analysis (TGA) experiments were conducted using a HITACHI STA200 thermogravimetric analyzer. The experiment was carried out in a nitrogen atmosphere, with a temperature range from 30 to 600 °C and a heating rate of 10 °C min^−1^. By comparing the thermogravimetric curves of the PEG, GmSP, empty carrier CP‐blank and the pheromone‐loaded carrier CP, the thermal stability and differences of the various samples during heating were analyzed.

### Mechanical Performance Characterization

The 3D‐printed samples were subjected to standard compression tests using an electronic universal testing machine, with a loading rate set to 0.5 mm min^−1^. Stress–strain curves were plotted, and the maximum compressive strength σ_max_ and elastic modulus *E* of the 3D‐printed carriers were further calculated. The specific calculation formulas are as follows:

(1) $$\sigma_{\max}=\frac{F_{\max}}{A}E=\frac{\Delta \sigma }{\Delta \varepsilon }$$

where σ_max_ is the maximum compressive strength, *F*_max_ is the maximum applied load, *A* is the cross‐sectional area of the sample; *E* is the elastic modulus, Δσ is the change in stress, and Δε is the change in strain.

### Tensile Test

The tensile test was conducted using a microcomputer‐controlled electronic universal testing machine (Model: CMT4503). The samples CP and CP‐SL‐Fe were printed into a dog bone shape with dimensions of 50 mm (length) × 10 mm (width) × 2 mm (height). All tests were performed under displacement control mode at a rate of 10 mm·min^−1^.

### Ultraviolet‐Visible‐Near Infrared Diffuse Reflectance Spectroscopy Characterization

The surface of the 3D‐printed samples was tested for diffuse reflectance using a Shimadzu UV‐3600 UV/Visible/Near‐Infrared spectrophotometer, with a testing wavelength range from 200 to 800 nm. The reflectance (R%) curves of the 3D‐printed samples before and after modification were obtained through the tests.

### Wettability Characterization

This section studies the wettability of the carrier surface before and after modification. Observations and measurements were performed using an OCA 20 contact angle goniometer (Data Physics). During each measurement, 2 µL of water was placed on the surface of the two types of 3D‐printed samples and allowed to settle for 15 s. All measurements were repeated three times.

### Degradability Study of 3D‐Printed Carriers

To assess and compare the degradability of the 3D‐printed carriers, CP and CP‐SL‐Fe and commercial rubber carriers were buried in 10 cm deep natural soil, which was taken from Pinggu District, Beijing, China (40.04917° N, 117.01972° E). The weight of the samples was recorded every 30 days, and the weight loss ratio was calculated. Three parallel samples were set up for each experiment.

### Encapsulation and Release Performance Study

The 3D‐printed pheromone‐loaded samples were placed in afume hood at room temperature (25 ± 2 °C, well‐ventilated with no directed airflow). Samples were taken at different time points, and the residual pheromone content was analyzed using a gas chromatograph (GC), with three parallel samples in each group. GC analysis was performed using an Agilent Technologies 7890A gas chromatograph equipped with a DB‐5 capillary column (30 m × 0.32 mm; film thickness 0.25 µm). Specific parameters: column temperature set to 60 °C, with a temperature ramp of 10 °C min^−1^ to 220 °C; both the injector and detector temperatures were set to 220 °C; the carrier gas was nitrogen at a flow rate of 2 mL min^−1^, with a split ratio of 20:1. The sample volume was 1 µL.

### Field Application

Field trapping experiments were conducted from August to September 2024 in Pinggu District, Beijing. 3D‐printed samples and commercial rubber stopper samples were installed in matching boat‐shaped traps, with traps spaced 10 meters apart. The number of catches was investigated weekly, and the sticky boards were replaced while the trap positions were changed to minimize location‐related errors. The field survey lasted for 6 weeks, and throughout the experiment, neither the samples nor the boat‐shaped traps were replaced. The effective pheromone content in each sample was maintained at 1 mg, with three replicates for each treatment.

### Recyclability Study

This study investigates the recyclability and reusability of 3D‐printed carriers after field use. First, the recycled 3D‐printed carriers were physically ground into powder. Then, a certain amount of pheromone was fully dissolved in a mixed solvent of acetone and dimethylformamide (4:1, v/v). Next, 15% (w/w) of the recycled powder was added to the mixed solvent and mechanically stirred at 800 rpm for 5 min to prepare a pheromone‐loaded ink for reprint, which was then printed and formed into new carriers, named rCP‐SL‐Fe. Repeat the above operation on rCP‐SL‐Fe to obtain the carrier r‐rCP‐SL‐Fe for three iterations. To verify the effectiveness of this recycling strategy, the surface morphology, pheromone encapsulation efficiency, Attenuated Total Reflectance Fourier Transform Infrared Spectroscopy (ATR‐FTIR), mechanical properties, and wettability of the secondary printed carriers were systematically evaluated. The printing process and parameters were consistent with the previous methods. Electroantennogram (EAG) recording was performed using an IDAC2 dual‐channel data acquisition controller system. The tips of both antennae from a live male Grapholita molesta moth were slightly trimmed and mounted between fork electrodes using a capillary tube, with the recording electrode connected to the IDAC2 system. The stimuli consisted of CP‐SL‐Fe, rCP‐SL‐Fe, and r‐rCP‐SL‐Fe (each loaded with 0.5 mg of pheromone), using a blank carrier (CP‐SL‐Fe‐blank) as the control. Each sample was tested with a minimum of five replicates. The EAG response value for each sample was calculated as the difference between the signals recorded for the sample and the control.

### Statistical Analysis

All data consist of at least three repetitions, and the results are presented as mean ± standard deviation. The results were analyzed using ORIGIN 2018 (OriginLab Corporation, Northampton, MA, USA) and SPSS v26.0 (SPSS Statistics/ IBM, Armonk, NY, USA).

## Conflict of Interest

The authors declare no conflict of interest.

## Author Contributions

T.W. and W.S. contributed equally to this paper. T.W., W.S., F.Z., and L.C. performed conceptualization; T.W., W.S., F.Z., W.L., F.R.W., Q.H., and L.C. performed methodology; T.W. and W.S. performed visualization; L.C. acquired Funding; F.Z., Q.H., and L.C. performed supervision; T.W., W.S., and L.C. wrote original draft; T.W., W.S., F.R.W., and L.C. wrote, reviewed, and edited the original manuscript.

## Supporting information



Supporting Information

## Data Availability

The data that support the findings of this study are available from the corresponding author upon reasonable request.
