# Supplementary material for: Degradable and Recyclable 3D‐Printed Pheromones Delivery System Reinforced by Metal Coordination Cross‐Linking for Efficient Pest Trapping
Source: Adv Sci (Weinh). 2025 Nov 29;13(9):e09712. doi: 10.1002/advs.202509712 (PMC12904024; doi:10.1002/advs.202509712)
Supplement: Supplementary file 1 — Supporting Information [file ADVS-13-e09712-s001.docx]

*Title Page*:

**Degradable and Recyclable 3D-Printed Pheromones Delivery System Reinforced by Metal Coordination Cross-Linking for Efficient Pest Trapping**

*Teng Wang^#^, Wenjie Shangguan^#^, Fang Zhang*, Wenlong Liang, Frederik R. Wurm*, Qiliang Huang and Lidong Cao**

T. Wang, W. Shangguan, Q. Huang, L. Cao

Key Laboratory of Integrated Pest Management in Crops

Institute of Plant Protection, Chinese Academy of Agricultural Sciences

Beijing 100193, P. R. China

1. mail: [caolidong@caas.cn](mailto:caolidong@caas.cn)
2. Wang, F. Zhang

College of Chemistry and Life Science, Beijing University of Technology

Beijing 100124, P. R. China

E-mail: [zhangfang2000@bjut.edu.cn](mailto:zhangfang2000@bjut.edu.cn)

W. Liang, F. R. Wurm

Sustainable Polymer Chemistry, Department of Molecules and Materials

MESA+Institute for Nanotechnology

Faculty of Science and Technology

Universiteit Twente

PO Box 217, Enschede 7500 AE, The Netherlands

E-mail: frederik.wurm@utwente.nl

#These authors contributed equally to this paper.

**Supporting Information**

**Table S1.** Surface roughness parameters (Rq and Ra) for CP and CP-SL-Fe.

| **Sample** | **Parameter** | **Mean (nm)** | **Range (nm)** |
| --- | --- | --- | --- |
| **CP** | **Rq** | **32.0** | **14.1** |
|  | **Ra** | **24.6** | **11.8** |
| **CP-SL-Fe** | **Rq** | **57.1** | **40.1** |
|  | **Ra** | **45.6** | **34.8** |

Ra: arithmetic average roughness; Rq: root mean square roughness.

**Table S2.** DMF residual ratio in CP and CP-wet samples.​

| **Sample** | **Mean ± SD (wt%)** | **N (Replicates)** |
| --- | --- | --- |
| **CP** | **0.27 ± 0.05** | **3** |
| **CP-wet** | **27.34 ± 1.32%** | **3** |

Values are presented as mean ± standard deviation. wt%: weight percentage.

**Table S3.** Zero-order, First-order, Higuchi, and Ritger-Peppas model fitting results of CP and CP-SL-Fe carriers.

| **Sample** | **Model** | **R^2^** |
| --- | --- | --- |
| CP | Zero-order | 0.95096 |
|  | First-order | 0.98396 |
|  | Higuchi | 0.98202 |
|  | Ritger-Peppas | 0.96967 |
| CP-SL-Fe | Zero-order | 0.67484 |
|  | First-order | 0.8725 |
|  | Higuchi | 0.83729 |
|  | Ritger-Peppas | 0.77611 |


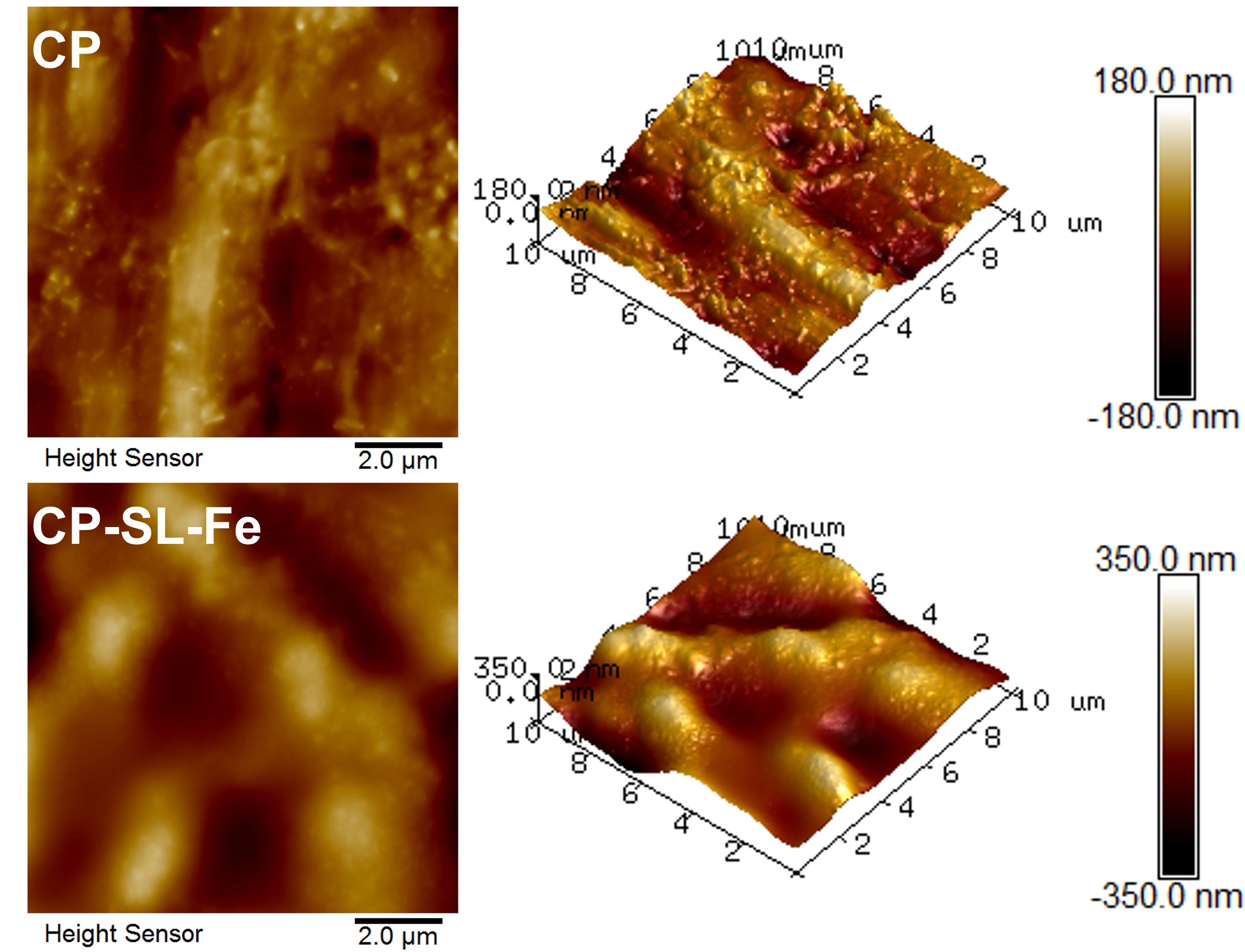


**Figure S1.** AFM three-dimensional topography images of CP and CP-SL-Fe.


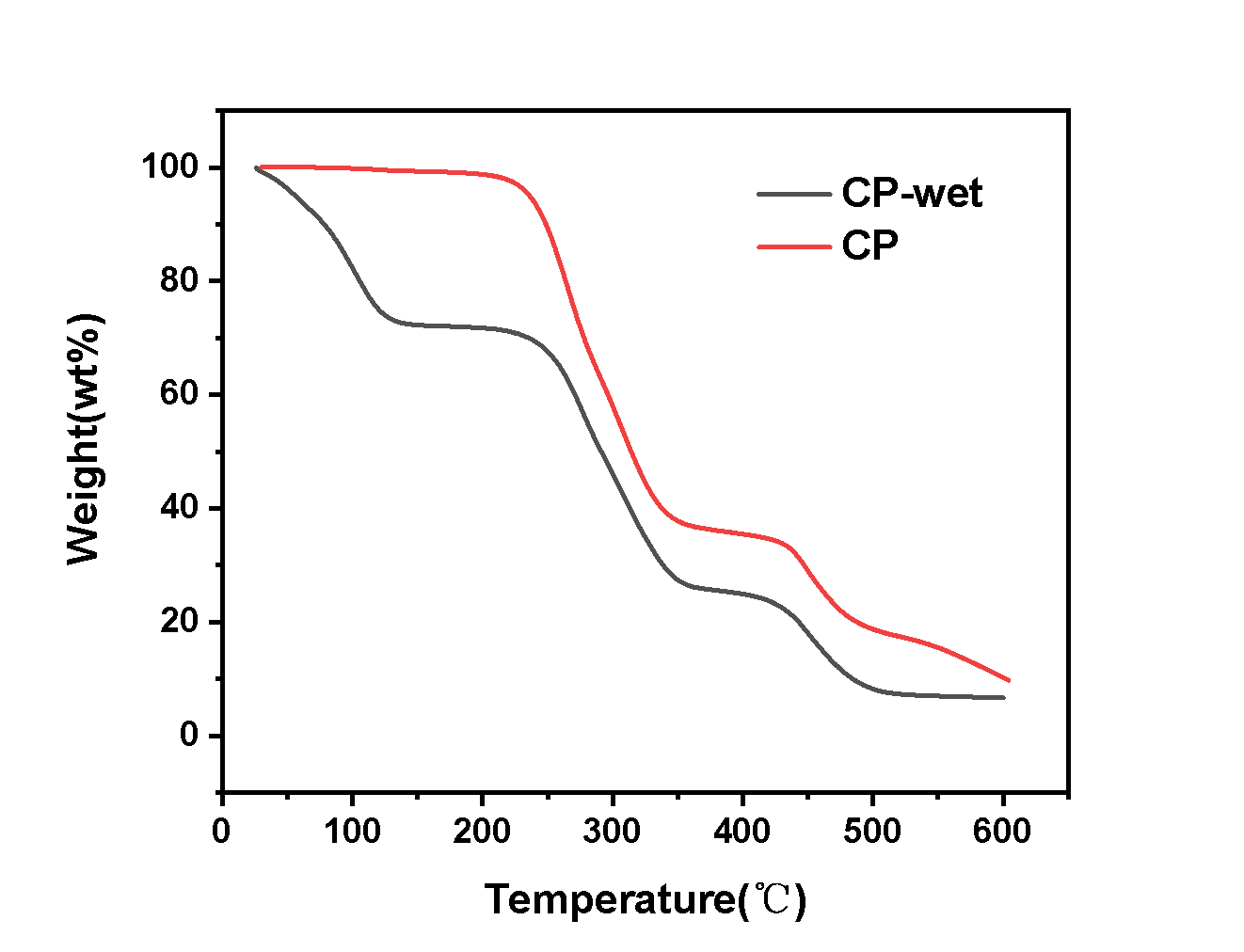


**Figure S2.** TGA thermal weight loss curve of CP-wet and CP.


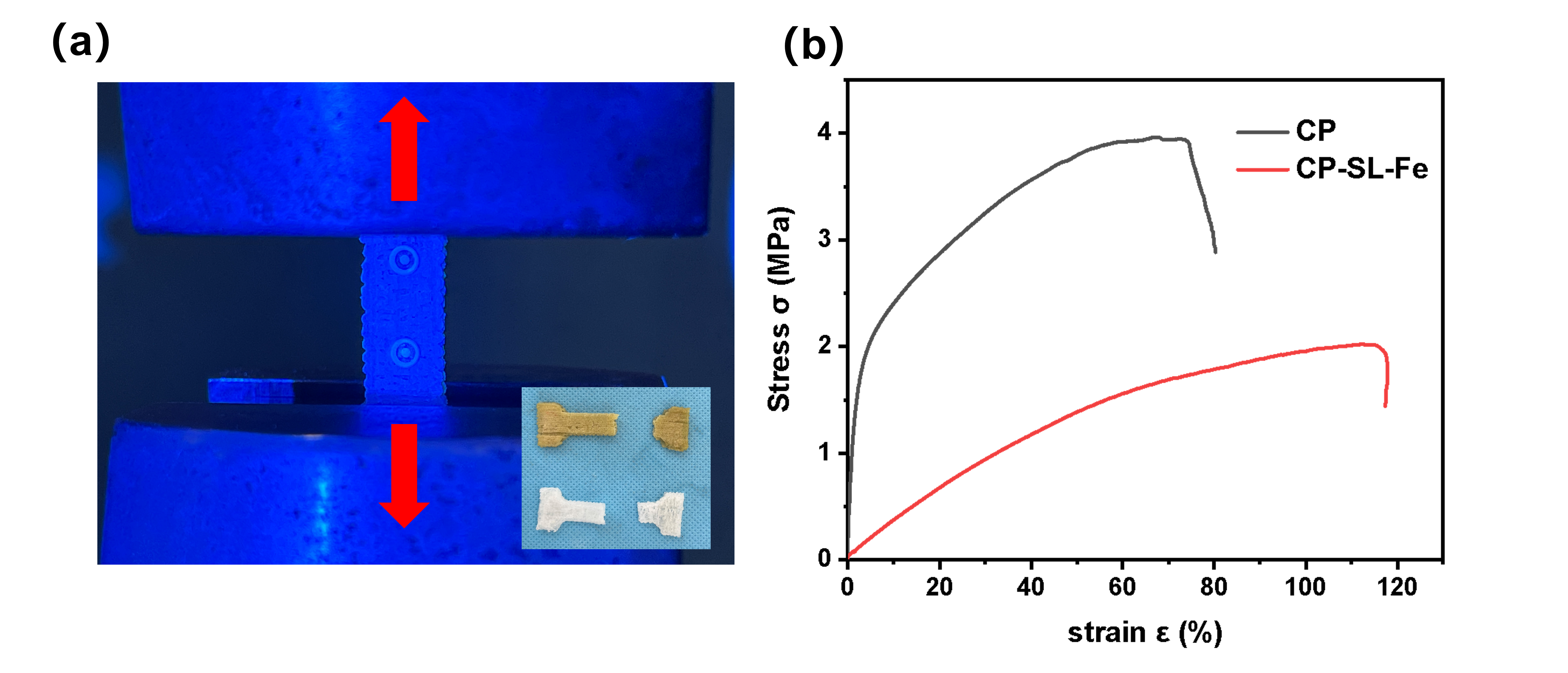


**Figure S3.** (a) Tensile test schematic and (b) Stress-strain curve of CP and CP-SL-Fe.

**
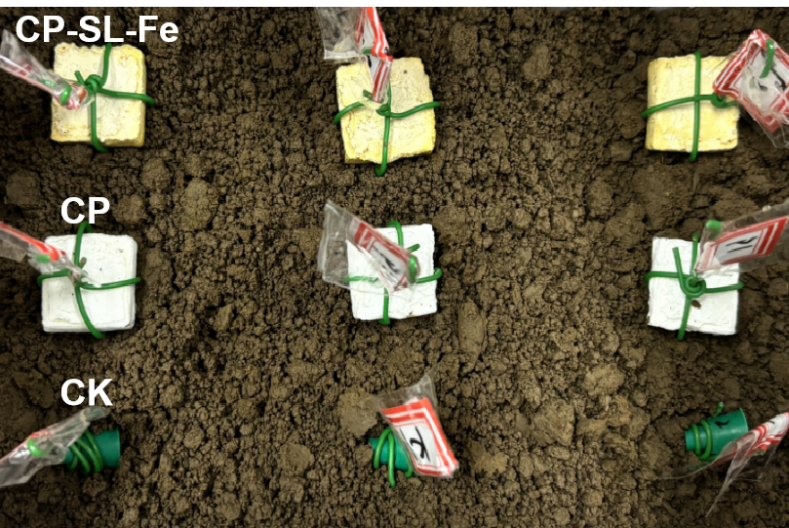
**

**Figure S4.** Photograph of the degradation experiment in soil for the CP-SL-Fe, CP, and CK samples, each with three replicates.

**
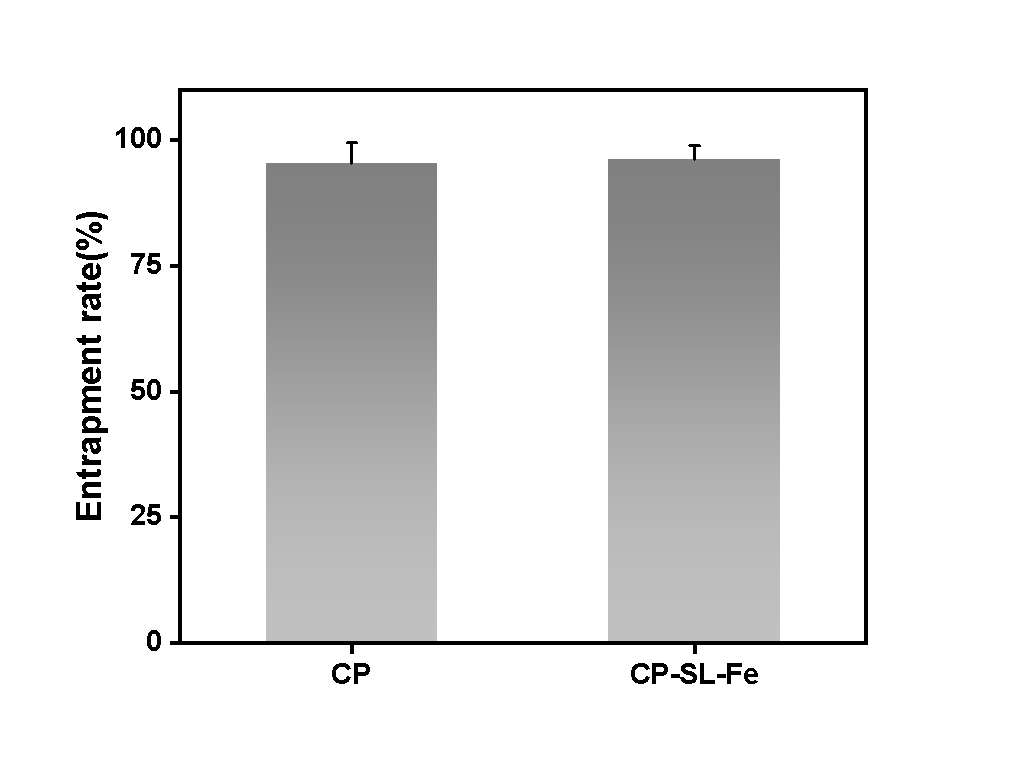
**

**Figure S5.** Comparison of pheromone encapsulation rates of CP and CP-SL-Fe.


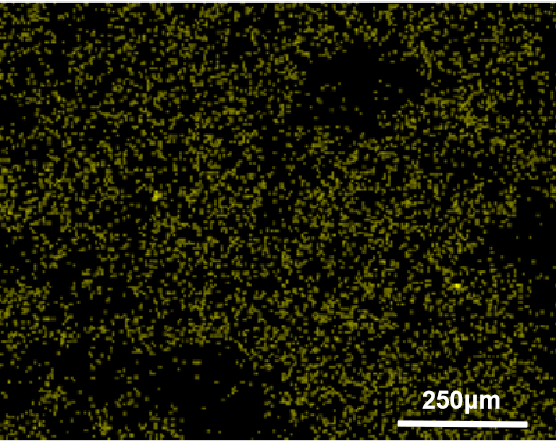


**Figure S6.** EDS mapping spectra of Fe in rCP-SL-Fe.


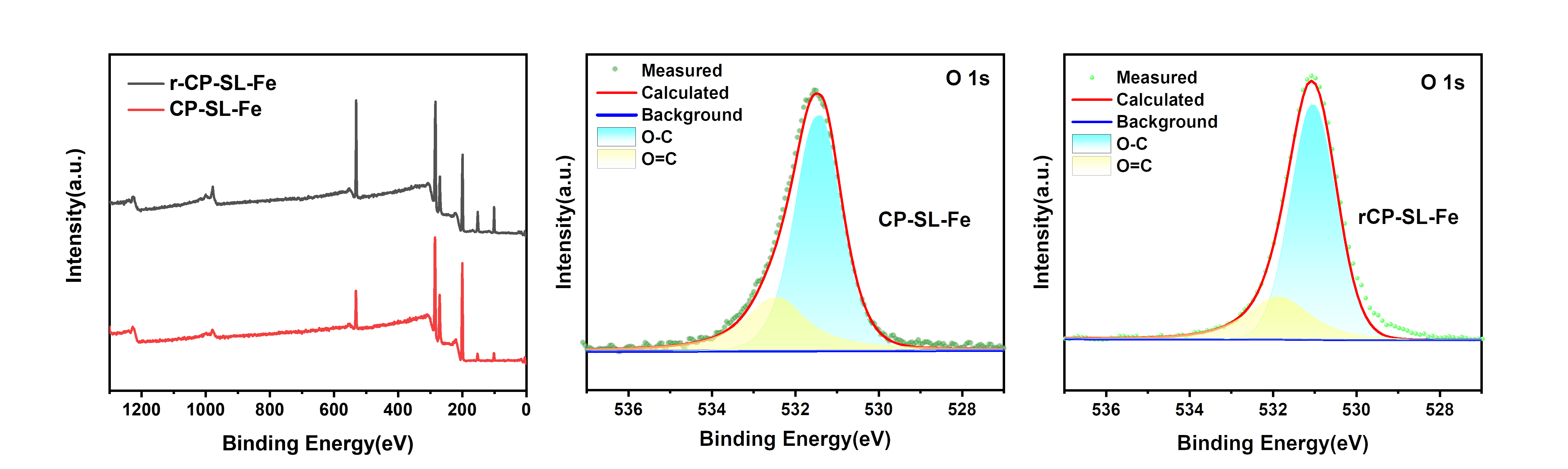


**Figure S7.** O1s XPS spectra of CP-SL-Fe and rCP-SL-Fe.


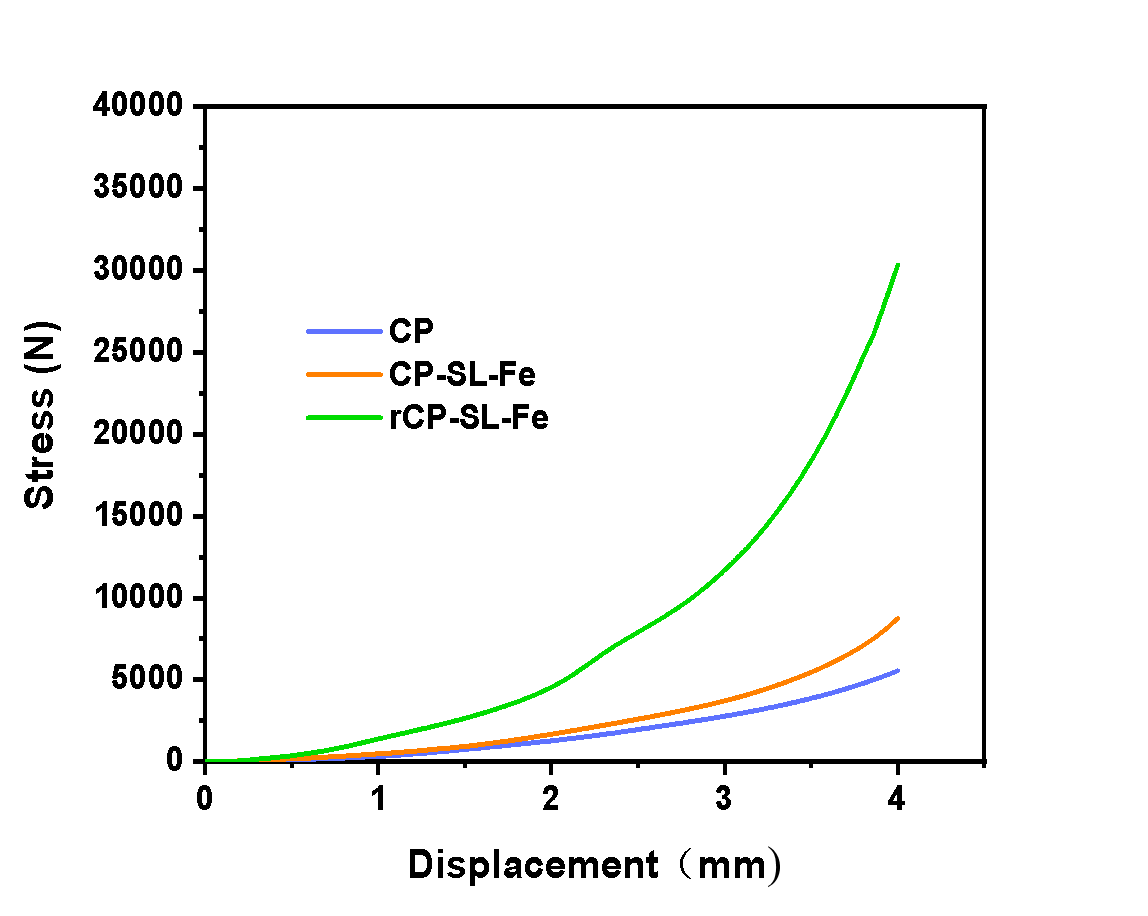


**Figure S8.** Force-displacement curve of CP, CP-SL-Fe and rCP-SL-Fe.


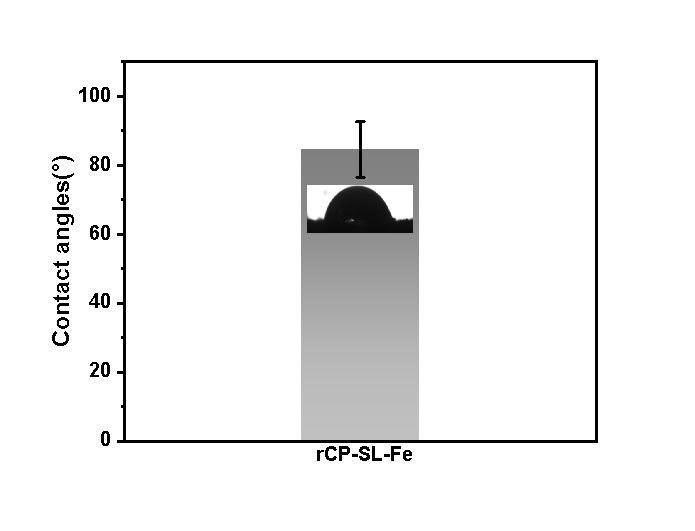


**Figure S9.** Water contact angle of rCP-SL-Fe.


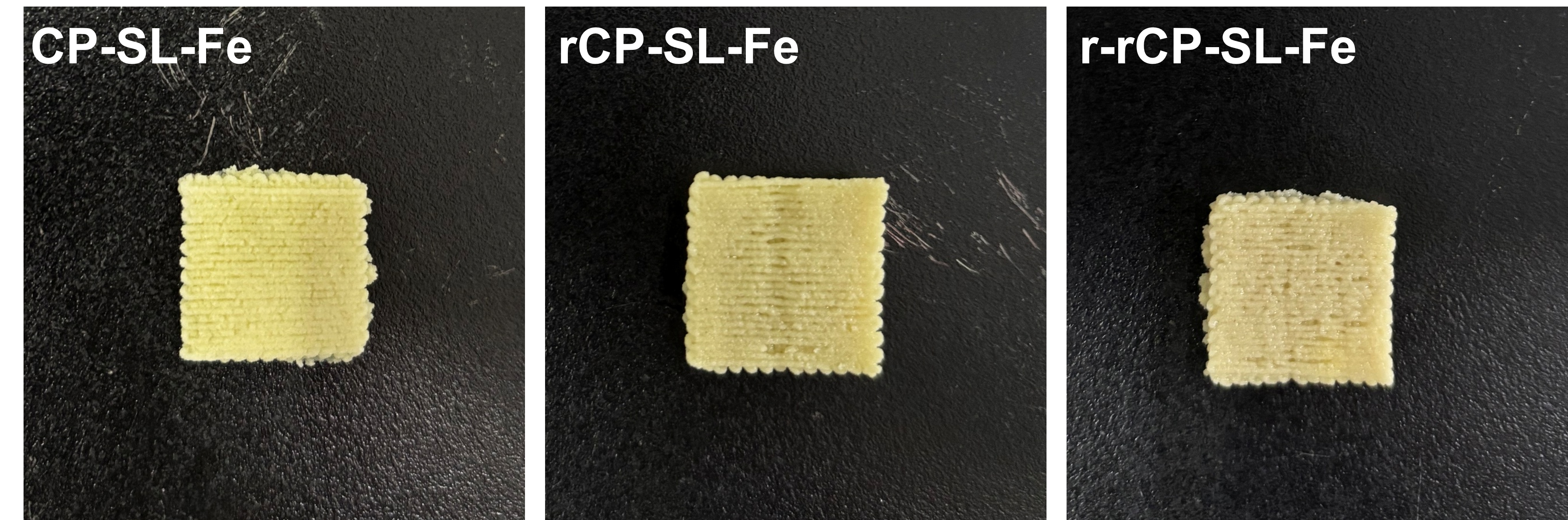


**Figure S10.** Photographs of the 3D-printed CP-SL-Fe, rCP-SL-Fe, and r-rCP-SL-Fe samples.


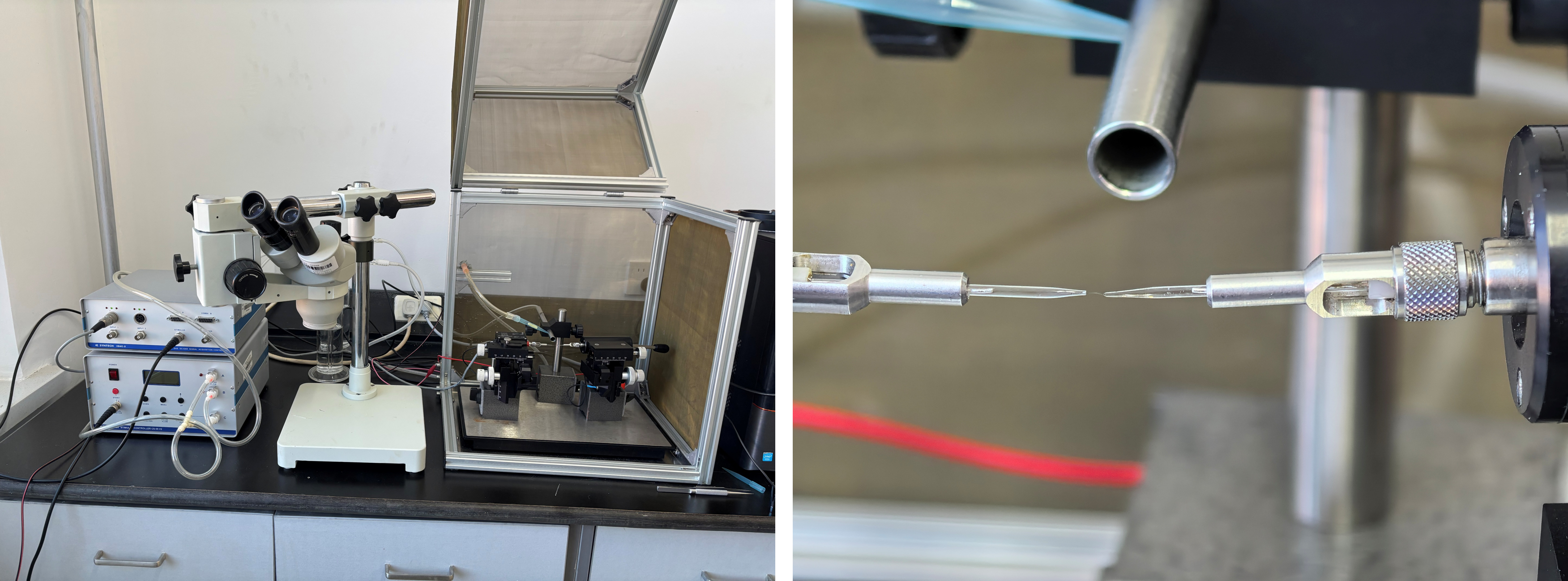


**Figure S11.** Diagram of the EAG experimental setup.


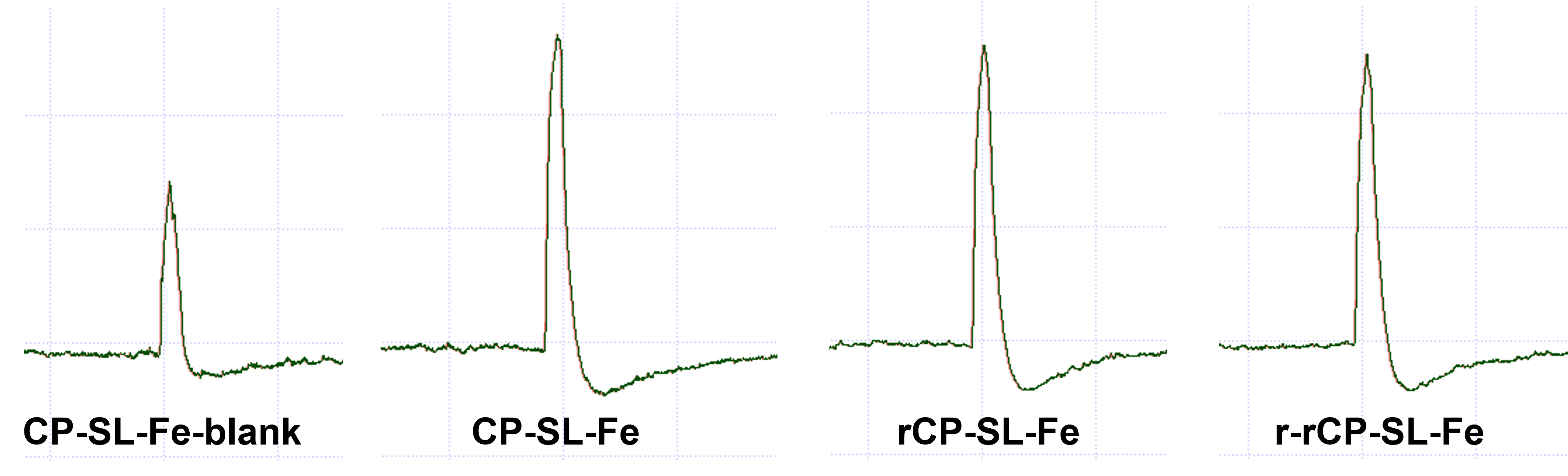


**Figure S12.** EAG responses of samples CP-SL-Fe, rCP-SL-Fe, and r-rCP-SL-Fe (referenced against CP-SL-Fe-blank).


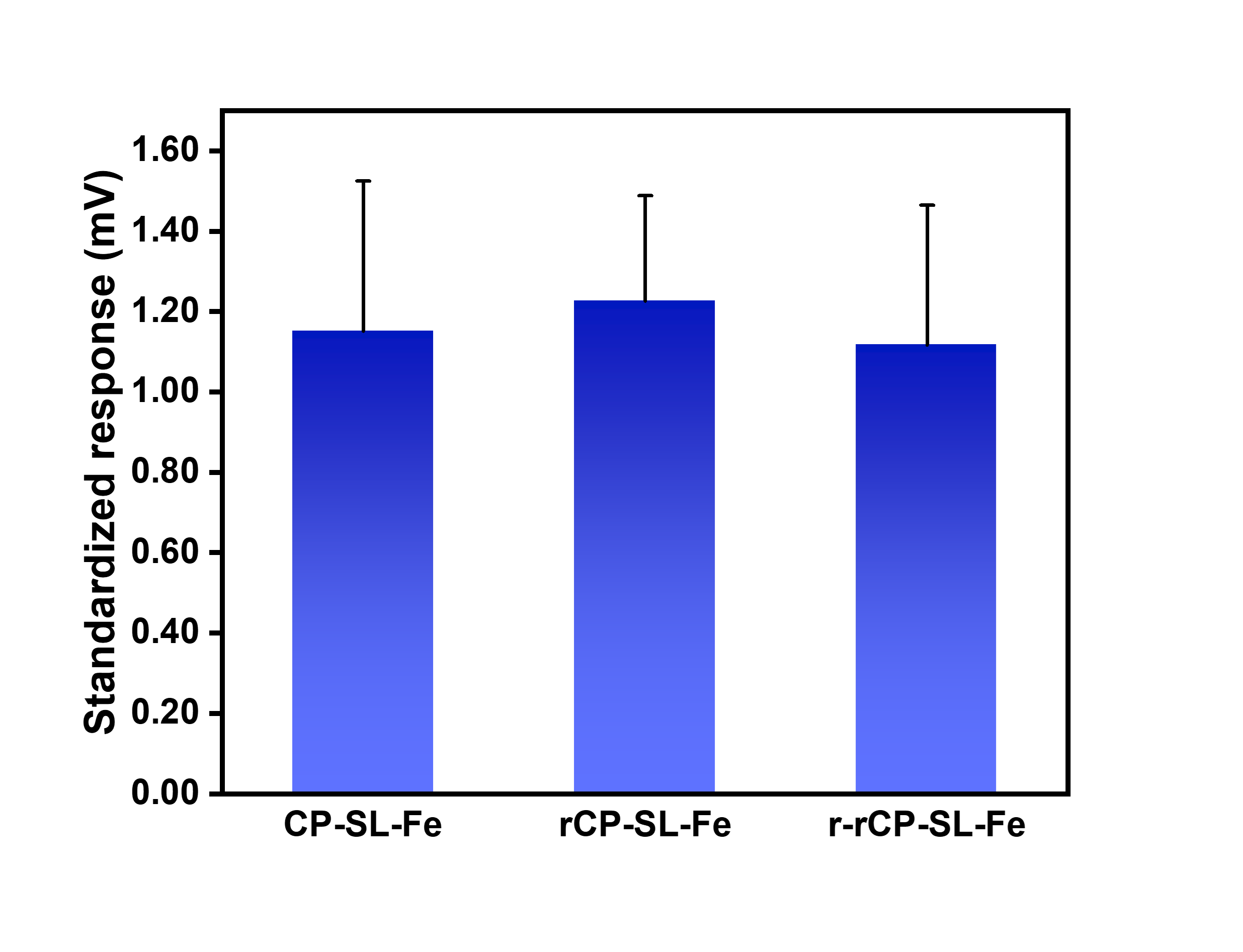


**Figure S13.** Normalized EAG response values of CP-SL-Fe, rCP-SL-Fe, and r-rCP-SL-Fe.
